# Engineering Bonding Types and Macromeric Cross-Linkers in the Network To Modulate the Structures and Properties of Polydextran/Chitosan Hybrid Hydrogels

**DOI:** 10.1021/acs.biomac.5c00677

**Published:** 2025-08-08

**Authors:** Pei-Han Lin, Tzu-Ying Wang, Yi-Cheun Yeh

**Affiliations:** Institute of Polymer Science and Engineering, 33561 National Taiwan University, Taipei 10617, Taiwan

## Abstract

Hybrid hydrogels are versatile biomaterials, and cross-linkers are the key for tailoring their characteristics. However, the bonding types in the network and the hydrophobic chain lengths of cross-linkers remain underexplored. This study introduces formylbenzoic acid-modified PEG (PEG-FA), which further reacts with adipic acid (AA), sebacic acid (SA), and tetradecanedioic acid (TDA) to create cross-linkers with varying hydrophobic segments. PEG-FA and its derivatives are used as aldehyde-functionalized macromeric cross-linkers to react with polydextran hydrazide (PDH) and quaternized chitosan (QCS) to form PDH/QCS hybrid hydrogels through dynamic interactions. The results indicate that increasing the hydrazone/imine ratio improves the strength and stability of the network. Notably, TDA-PEG-FA cross-linkers grant hydrogels with smaller pores and better mechanical strength. Overall, the structures and properties of PDH/QCS hydrogels can be tailored by selecting bonding types and designing macromeric cross-linkers in the network, enabling the customization of hydrogel performance for advanced applications.

## Graphical abstract

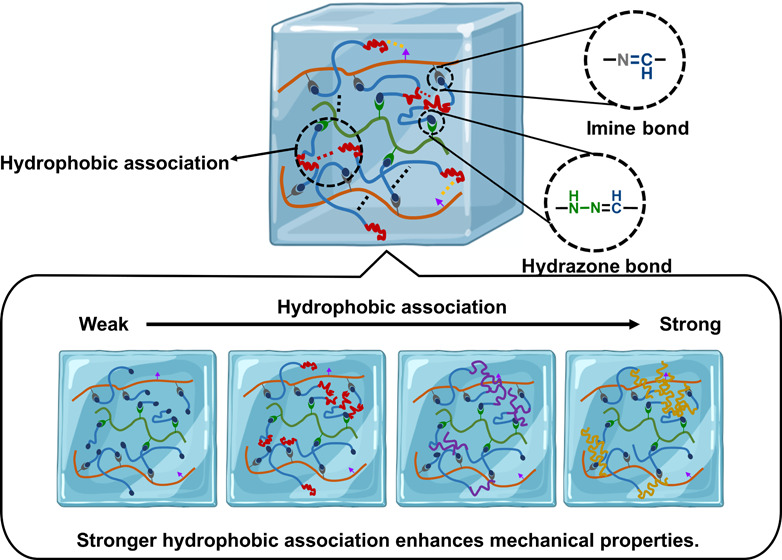

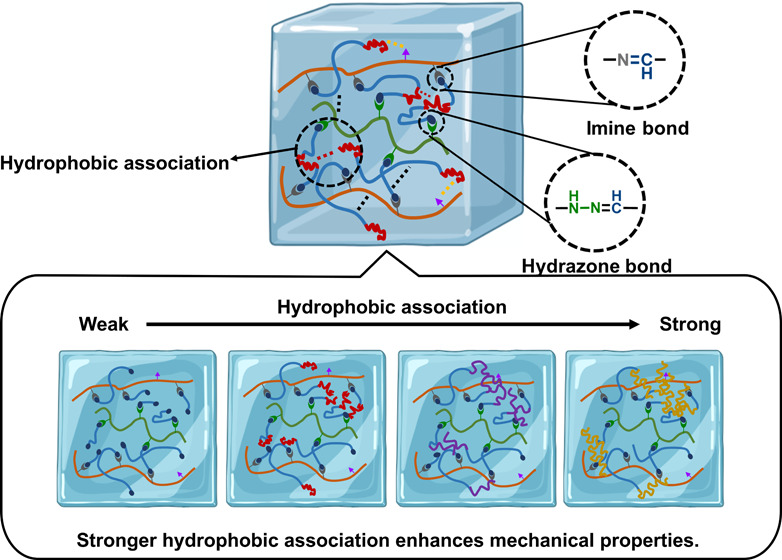

## 1 Introduction

Hybrid hydrogels have become promising biomaterials that integrate the unique features of multiple polymeric materials into a single system with tailorable mechanical, chemical, and biological properties.^1^ Several cross-linking approaches have been utilized to fabricate hybrid hydrogels, including noncovalent (e.g., hydrogen bond,^2^ hydrophobic interactions,^3^ and electrostatic interactions^4^), dynamic covalent (e.g., imine bond,^5^ hydrazone bond,^6^ and disulfide bond^7^), and covalent interactions.^8^ For example, Zhang et al. synthesized hybrid hydrogels by combining the two polymers of carboxymethyl chitosan and hyaluronic acid through imine cross-linking, where the chitosan not only increased the mechanical properties and biocompatibility of the hydrogel but also provided the hydrogel with antibacterial properties.^9^ Andrabi et al. fabricated the gelatin/dextran hybrid hydrogels through imine bonds between the amines of gelatin and aldehydes of oxidized dextran.^10^ They further incorporated curcumin and cerium oxide into the gelatin/dextran hybrid hydrogels to form a dressing that can slowly release curcumin into the wounds. Shen et al. developed a hydrogel composed of gallic acid-modified chitosan and oxidized dextran, forming a network through imine bonds and hydrogen bonds to enhance wound healing by eliminating reactive oxygen species in combined radiation and burn injuries.^11^

Using cross-linkers to connect multiple polymers to construct a hybrid network provides a simple and straightforward strategy to modulate the structures and properties of the hydrogels. For example, Khodami et al. employed iron­(III) (Fe^3+^) ions as cross-linkers to form coordination bonds with carboxyl groups on poly­(acrylic acid) and natural alginate, providing the hydrogel with mechanical strength and self-healing ability as well as enhanced electrical conductivity.^12^ On the other hand, Ren et al. used glutaraldehyde to fabricate a biodegradable and recyclable gelatin/chitosan hydrogel with enhanced mechanical strength and stability.^13^ In particular, macromer is another type of attractive cross-linker for hybrid hydrogel formation. For instance, Pruksawan et al. utilized polyhedral oligomeric silsesquioxane (POSS)-grafted acrylated polyethylene glycol (PEG) as a macro-cross-linker to achieve uniform connections in polyacrylamide hydrogels.^14^ The acrylate groups provided multiple interaction points, and the long PEG chains introduced physical entanglements, significantly improving the toughness and elongation of the hydrogel network. While there is an increasing interest in hybrid hydrogels, research on integrating different types of chemical cross-links and varying the hydrophobic chain lengths of cross-linkers within the hybrid network is still lacking.

Here, we hypothesize that macromeric cross-linkers can be engineered to connect each polymer through distinct reactions to increase the diversity of the hybrid hydrogel network. Particularly, macromeric cross-linkers with differing lengths of hydrophobic segments can be utilized to create various extents of hydrophobic domains within the hydrogel network, influencing the characteristics and performance of the hybrid hydrogels through the microphase separation.

In this study, polydextran and chitosan were selected as the polymeric matrix to construct hybrid hydrogels, with functionalized polyethylene glycol (PEG) designed to spontaneously cross-link these polymers through dynamic bonds (Scheme 1). Specifically, polydextran hydrazide (PDH) and quaternized chitosan (QCS) were prepared to present hydrazide and amine groups in the polymer structure, respectively. Additionally, formylbenzoic acid-modified polyethylene glycol (PEG-FA) with aldehyde groups was synthesized and also further reacted with dicarboxylic acids (i.e., adipic acid (AA), sebacic acid (SA), and tetradecanedioic acid (TDA)) to result in three types of dicarboxylic acid-incorporated PEG-FA derivatives with varying hydrophobic segments (i.e., AA-PEG-FA, SA-PEG-FA, and TDA-PEG-FA). These PEG-FA and PEG-FA derivatives were then combined with PDH and/or QCS to fabricate hydrogels through dynamic interactions (i.e., imine bonds, hydrazone bonds, hydrophobic interactions, electrostatic interactions, and hydrogen bonds), generating 20 types of hydrogels. The roles of the bonding types in the network and the hydrophobic segment lengths in the macromeric cross-linkers on the structures and properties of the hybrid hydrogels were systematically investigated.

**Scheme 1:**
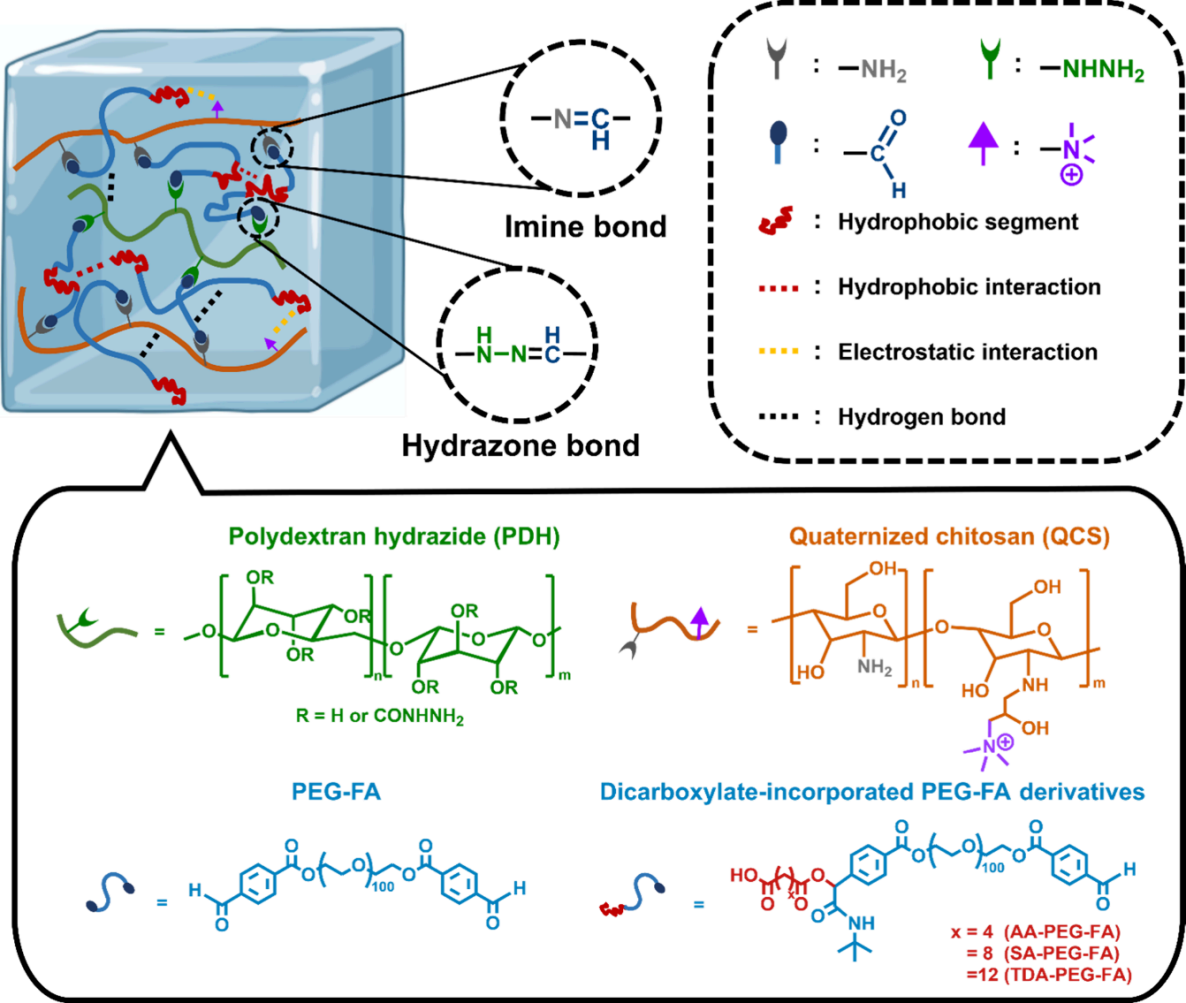
Schematic Illustration of the Materials and the Cross-Linking Chemistry in the Hydrogel Network

## 2 Materials and Methods

### 2.1 Materials

Polyethylene glycol (4 kDa), polydextran (40 kDa), hydrazine hydrate, and ninhydrin were purchased from Alfa Aesar. Sebacic acid and chitosan (low molecular weight, 50–190 kDa) were purchased from Sigma-Aldrich. Adipic acid, glycidyltrimethylammonium chloride, and tetradecanedioic acid were purchased from TCI. 1-Ethyl-3-(3-dimethylaminopropyl) carbodiimide hydrochloride, 4-dimethylaminopyridine, and 4-formylbenzoic acid were purchased from Fluorochem. Methanol, dimethylformamide, and dimethyl sulfoxide were purchased from Duksan. Acetic acid was purchased from Honeywell Fluka. 1,1’-Carbonyldiimidazole and tert-butyl isocyanide were purchased from Macklin. The polydimethylsiloxane (PDMS) mold for tensile testing is made by mixing Part A (the base agent) and Part B (the curing agent), both of which are purchased from Gelest. In cellular studies, Dulbecco’s Modified Eagle Medium (DMEM), fetal bovine serum (FBS), penicillin/streptomycin, 4-(2-hydroxyethyl)-1-piperazine­ethanesulfonic acid (HEPES), alamarBlue assay, and live–dead cell viability assay kit were purchased from ThermoFisher.

### 2.2 Instruments

The chemical characterization of polymers was conducted by ^1^H nuclear magnetic resonance (NMR) (AVIII HD 400, Bruker) and Fourier transform infrared spectroscopy (FTIR) (Spectrum two, PerkinElmer). The molecular weight of polymers was measured by gel permeation chromatography (GPC) (SUPER CO-150, Enshine), and the GPC was equipped with the Waters 2414 refractive index detector (RI) and TSK gel G5000PW column. The hydrogels in pore size characterization were lyophilized first by a freeze-dryer (FDM-2, UNISS) at −80 °C and 10 mTorr. The pore sizes of hydrogels were examined by scanning electron microscopy (SEM) (TM-3000 tabletop SEM, Hitachi) and microcomputed tomography (Micro-CT) (1076, Skyscan). The SEM images were analyzed by ImageJ. The compressive test, cyclic compression test, and tensile test were evaluated by a material testing system (AGS 500N, Shimadzu). The rheology analyses were conducted by rheometer (HR-2 system, TA Instrument). The thermal stability was tested by differential scanning calorimetry (DSC) (Q-20, TA Instruments). The conductivity of hydrogels was measured by electrical impedance spectroscopy (PGSTAT302, Metrohm). The resistance value change was monitored by a multimeter (DMM6500, Keithley). The fluorescence intensity in the cellular viability test was measured by a microplate reader (Synergy H1, BioTek). The fluorescent-stained cells were observed by an automated fluorescence microscope (Nikon Ti2E).

### 2.3 Synthesis of Polydextran Hydrazide (PDH)

Polydextran hydrazide (PDH) was prepared based on the reported procedure.^15^ Polydextran (0.5 g) was dissolved in dimethyl sulfoxide (DMSO, 20 mL) at 80 °C for 15 min, and the solution was cooled down to room temperature. After that, 1,1’-carbonyldiimidazole (1 g, 6.17 mmol) was added to the solution at room temperature for 24 h, and hydrazine hydrate (80 wt %, 3.2 mL, 50.18 mmol) was dropped into the mixture, reacting for 24 h at room temperature. The mixture was dialyzed against deionized water using a dialysis membrane (MWCO 3500 Da, MFPI) for 3 days and lyophilized.

### 2.4 Synthesis of Quaternized Chitosan (QCS)

Quaternized chitosan (QCS) was synthesized according to the previous report.^16^ Chitosan (2 g) was suspended in an acetic acid solution (0.5% v/v, 200 mL) and stirred until fully dissolved. Glycidyl trimethylammonium chloride (GTMAC, 10 mL, 78.1 mmol) was then added dropwise to the chitosan solution under continuous stirring at 55 °C for 18 h. The resulting mixture was filtered and dialyzed against deionized water using a dialysis membrane (MWCO 12–14 kDa, MFPI) for 3 days. The final QCS product was obtained through lyophilization, and the percentage of GTMAC modification on chitosan was calculated using a ninhydrin test.^17^

### 2.5 Syntheses of PEG-FA and Dicarboxylic Acid-Incorporated PEG-FA Derivatives

Polyethylene glycol (PEG, 5 g, 1.25 mmol), formylbenzoic acid (FA, 3.75 g, 20 mmol), 1-ethyl-3-(3-dimethylaminopropyl)­carbodiimide (EDC, 3.88 g, 25 mmol), and dimethylaminopyridine (DMAP, 0.61 g, 5 mmol) were combined in dimethylformamide (DMF, 30 mL) and stirred at 45 °C for 72 h. The mixture was subsequently dialyzed against deionized water using a dialysis membrane (MWCO 3500 Da, MFPI) for 1 day and lyophilized to obtain the final product.^18^

PEG-FA (2.10 g, 0.5 mmol) and tert-butyl isocyanide (BI, 0.17 g, 2 mmol) were dissolved in methanol (20 mL) and reacted with various dicarboxylic acids (i.e., adipic acid (AA, 0.07 g, 0.5 mmol), sebacic acid (SA, 0.1 g, 0.5 mmol), or tetradecanedioic acid (TDA, 0.13 g, 0.5 mmol)) via Passerini reaction.^19^ The mixture was stirred at 45 °C for 48 h, dialyzed using a membrane (MWCO 3500 Da, MFPI), and lyophilized to yield the product.

The aldehyde modification ratio of the PEG-FA and dicarboxylic acid-incorporated PEG-FA derivatives was determined by the integration in the NMR spectra. The protons on PEG (-CH_2_CH_2_O-) at 3.5–3.62 ppm were set to be 400 (based on PEG-4000 with ∼100 repeat units). The integrated value of aldehyde protons (-CHO) at 10.01 ppm should be 2 if fully modified. Thus, the modification percentage was calculated by using the following equation:

$$\mathrm{modification percentage}=\frac{\mathrm{integrated value of aldehyde peak}}{2}\times 100\%$$

### 2.6 Preparation of Hydrogels

The solutions of PEG-FA (10 wt %), AA-PEG-FA (19.9 wt %), SA-PEG-FA (21.1 wt %), TDA-PEG-FA (20 wt %), QCS (5 wt %), and PDH (5.1 wt %) were prepared in phosphate-buffered saline (PBS, pH 7.4). The aldehyde contents of PEG-FA, AA-PEG-FA, SA-PEG-FA, and TDA-PEG-FA were determined via NMR spectroscopy to be 0.49, 0.28, 0.22, and 0.25 mmol/g, respectively. The amine content in QCS was quantified as 0.50 mmol/g using the ninhydrin assay. Similarly, the hydrazide content in PDH, determined by the ninhydrin assay, was found to be 0.50 mmol/g. Hydrogels were synthesized by mixing equal volumes of PEG-FA (10 wt %), AA-PEG-FA (19.9 wt %), SA-PEG-FA (21.1 wt %), and TDA-PEG-FA (20 wt %). The ratio of PDH (5.1 wt %) to QCS (5 wt %) was adjusted according to the NHNH_2_:NH_2_ molar ratio, with formulations prepared at ratios of 0:1 (PDH), 1:1 (P1Q1), 1:2 (P1Q2), 1:3 (P1Q3), and 0:1 (QCS). The volumes of PDH and QCS were adjusted to achieve the desired functional group ratios, ensuring that the total molar ratio of NHNH_2_ and NH_2_ to CHO was maintained at 1 across all hydrogels.

### 2.7 Microstructures, Compression, and Tensile Tests of Hydrogels

The hydrogel samples were freeze-dried to preserve their internal porous structure. After drying, the samples were placed into a scanning electron microscope (SEM) or a micro-computed tomography (micro-CT) system to observe and analyze the morphology and pore size of the hydrogel structures. Mechanical properties of hydrogels were evaluated by compression and tensile testing using the instrument of materials testing system installed with a 10 N load cell. The compression properties were measured at the crosshead speed of 5 mm/min, and the tensile testing was 1 mm/min. For the compression test, hydrogels were prepared in a cylinder shape with a 4.7 mm diameter and 5.2 mm height; for tensile testing, hydrogels were formed into dog-bone shaped polydimethylsiloxane (PDMS) molds (2.6 mm thick, 5.0 mm width at center). Force–displacement curves obtained from the machine were converted to stress–strain curves. Compressive modulus and tensile modulus were determined from the slope of the linear stress–strain curve between 10% and 20% strain.

### 2.8 Rheology Studies of Hydrogels

The rheological studies of the hydrogels were conducted by a rheometer with an 8 mm parallel plate. The hydrogels were placed at the center of the plate for the time sweep test. The storage modulus (*G*′) of hydrogels was monitored with a 1.0% strain at a frequency of 1 Hz and a temperature of 25 °C.

### 2.9 Adhesion Measurement of Hydrogels

The measurement of adhesive properties of hydrogels was performed on a materials testing system installed with a 500 N load cell at 1 mm/min crosshead speed. The porcine skins were cut into 30 mm × 10 mm sizes. The hydrogels were applied to porcine skin and covered an area of 10 mm × 10 mm. Then, the porcine skin pieces were pressed by a 1.35 kg book for 1 h. The porcine skin pieces sandwiched with hydrogels were put on the materials testing system to measure the shear strength of hydrogels to porcine skin.

### 2.10 Degradation of Hydrogels

The weight of lyophilized hydrogels (∼120 mg) was the dried weight (*w*_0_). The lyophilized hydrogels were soaked in PBS (1 mL) at 37 °C and the samples were put on a shaker with 100 r.p.m shaking speed. At the time points 1, 3, 5, and 7 days, the hydrogels were lyophilized, and evaluate the remaining weight (*w*_1_). The weight remaining ratio was calculated using the following equation:

$$\mathrm{weight remaining ratio}=\frac{w_{1}}{w_{0}}\times 100\%$$

### 2.11 Water Content of Hydrogels

The lyophilized hydrogels (∼120 mg) were immersed in the PBS solution (1 mL) for different periods. The initial weight of lyophilized hydrogels was the dried weight (*w*_*d*_), and the weight of immersed hydrogels after distinct time was wet weight (*w*_*s*_). The water content was calculated by the following equation.

$$\mathrm{water content}\left(\%\right)=\frac{w_{s}-w_{d}}{w_{s}}\times 100\%$$

### 2.12 Electrical Properties of Hydrogels

In the conductivity test, the hydrogels were cut into a cuboid of 15 mm in length, 5 mm in width, and 3 mm in thickness, and the copper tape was attached to both ends of the hydrogel as electrodes. The resistance was measured by electrical impedance spectroscopy in the frequency range from 10^–1^ Hz to 10^5^ Hz. The conductivity of hydrogels was confined by the equation^20^

$$\sigma =\frac{S}{R\times A}$$

in which σ (Ω^–1^ m^–1^) is the conductivity, S (m) is the length, R (Ω) is the measured resistance, and A (m^2^) is the cross-sectional area of the hydrogels. In detecting human motion, the hydrogels were cut into a 15 mm × 5 mm × 3 mm cuboid, and copper tape connected to the wires of a digital multimeter was attached to both ends of the hydrogel as electrodes. The detected hydrogel was placed at the joint of the wrist or finger. The relative resistance was calculated by the following equation.^21^

$$\frac{\Delta R}{R_{0}}=\frac{R-R_{0}}{R_{0}}\times 100\%$$

In this equation, ΔR represents the difference between real-time and initial resistance. The hydrogels in the resistance sensitivity test were cut into the same shape as the test of human motion detection and placed on the materials testing system installed with a 10 N load cell at a crosshead speed of 100 mm/min. In the electrical cyclic stability test, the hydrogels were placed on the materials testing system to stretch to 30% strain and returned to the original state for 100 cycles.

### 2.13 Cellular Study of Hydrogels

Mouse embryonic fibroblasts (MEFs) were isolated from C57BL/6N mice embryos and then immortalized after 15 passages. The cells were cultured at 37 °C in a 5% CO_2_ humidified incubator with Dulbecco’s Modified Eagle Medium (DMEM) supplemented with 10% fetal bovine serum (FBS), 1% penicillin/streptomycin, and 1% 4-(2-hydroxyethyl)-1-piperazineethanesulfonic acid (HEPES). The metabolic activity of MEFs was assessed using the alamarBlue assay, following the guidelines of the manufacturer. Cells were incubated with alamarBlue solution for 4 h. Cellular viability at each time point was measured using a microplate reader with an excitation wavelength of 530 nm and an emission wavelength of 590 nm. Results were normalized to the readings obtained on day 1 (D1) for each sample. For live/dead staining, MEFs (3 × 10^4^ cells/mL, 200 μL) were seeded in a 96-well plate and incubated with extract solutions obtained from the hydrogels. MEFs were identified by fluorescent staining of live cells as green (2 μM calcein-AM) and dead cells as red (4 μM ethidium homodimer-1). The stained cells were washed with PBS solution and observed by an automated fluorescence microscope.

### 2.14 Statistical Analysis

The experiments in this study were conducted with three replicates. Error bars were reported in figures as the standard deviation. The *t* test analysis was used to determine the statistical significance of differences in the data analysis. Significance was set at *p* < 0.05 with *, **, or *** referring to *p* < 0.05, 0.01, or 0.001, respectively.

## 3 Results

### 3.1 Syntheses and Characterizations of Polymers

Polydextran hydrazide (PDH) was synthesized through the two-step process in the Mitsunobu reaction.^15^ 1,1′-Carbonyldiimidazole was used as a leaving group to be coupled with the hydroxyl group of polydextran and form the imidazole carbonate intermediate, and then the intermediate was deprotected by hydrazine to form the carbazate group (Scheme 2a). Compared to the spectra of polydextran, the distinct peak for the protons of hydrazide appeared at 5.28 ppm in the ^1^H NMR spectrum of PDH (Figure S1a,b), and the C=O peak was detected at 1715 cm^–1^ in the FTIR spectrum of PDH (Figure S1c).

**Scheme 2:**
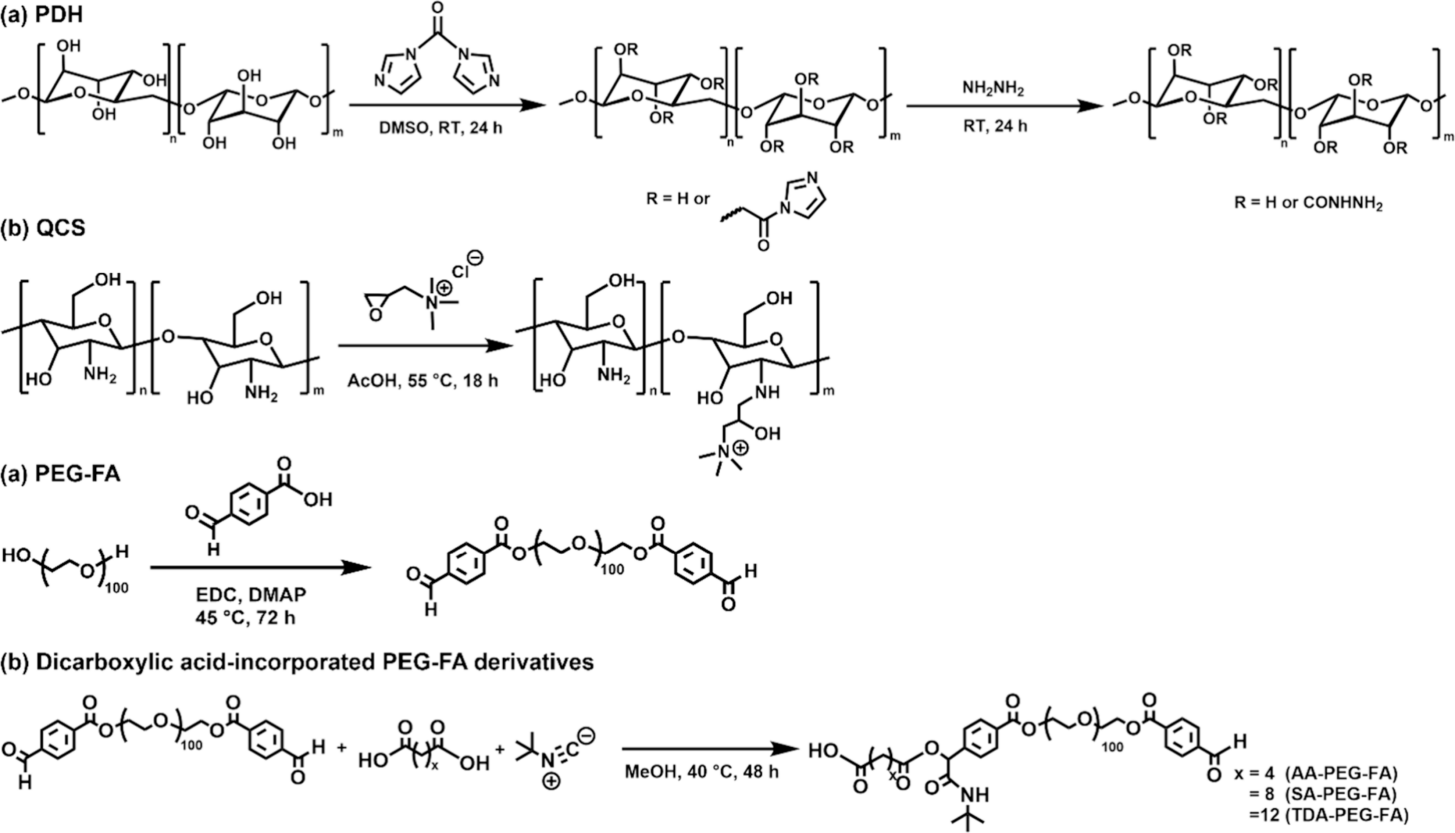
Synthetic Schemes of (a) PDH, (b) QCS, (c) PEG-FA, and (d) Dicarboxylic Acid-Incorporated PEG-FA Derivatives

Quaternized chitosan (QCS) was prepared by using chitosan to react with glycidyl trimethylammonium chloride (GTMAC) through a quaternization reaction (Scheme 2b). In the ^1^H NMR spectrum of QCS, the characteristic peak of the protons from the trimethylammonium (-N^+^(CH_3_)_3_) groups can be observed at 2.88 ppm (peak j) (Figure S2a). In the FTIR spectrum of QCS, the peak at 1480 cm^–1^ was attributed to an asymmetric angular bending of methyl groups in quaternary ammonium groups, and the peak at 1566 cm^–1^ corresponded to the N–H stretch of the remaining amines in QCS (Figure S2b). The synthesized QCS exhibited ∼86% substitution with GTMAC determined through the ninhydrin test (Figure S3).

Formylbenzoic acid-modified polyethylene glycol (PEG-FA) was synthesized via Steglich esterification (Scheme 2c).^22^ The formylbenzoic acid was activated with 1-ethyl-3-(3-dimethylaminopropyl)­carbodiimide (EDC) in the presence of 4-dimethylaminopyridine (DMAP), and then the activated acid reacted with the hydroxyl group on PEG to form a PEGylated ester, linking PEG to formylbenzoic acid under mild conditions. In the ^1^H NMR spectrum of PEG-FA macromer (Figure 1a, Figure S4), the peaks in the range of 3.50 ∼3.72 ppm (peak a, b) corresponded to the methylene protons (−COCH_2_CH_2_O−) of PEG segments and the peak at 10.02 ppm (peak c) was recognized as the proton of the aldehyde group. The modification percentage of aldehyde in the PEG-FA structure was calculated using ^1^H NMR spectrum, showing ∼98% of aldehyde content (0.49 mmol/g). PEG-FA was further incorporated with dicarboxylic acids with varying hydrophobic chain lengths through the Passerini reaction (Scheme 2d).^23^ The carbonyl carbon of the aldehyde group in PEG-FA was first attacked by the isocyanide to generate an intermediate. Subsequently, the dicarboxylic acid reacted with the intermediate and formed an α-acyloxy amide product through Mumm’s rearrangement.

**Figure 1:**
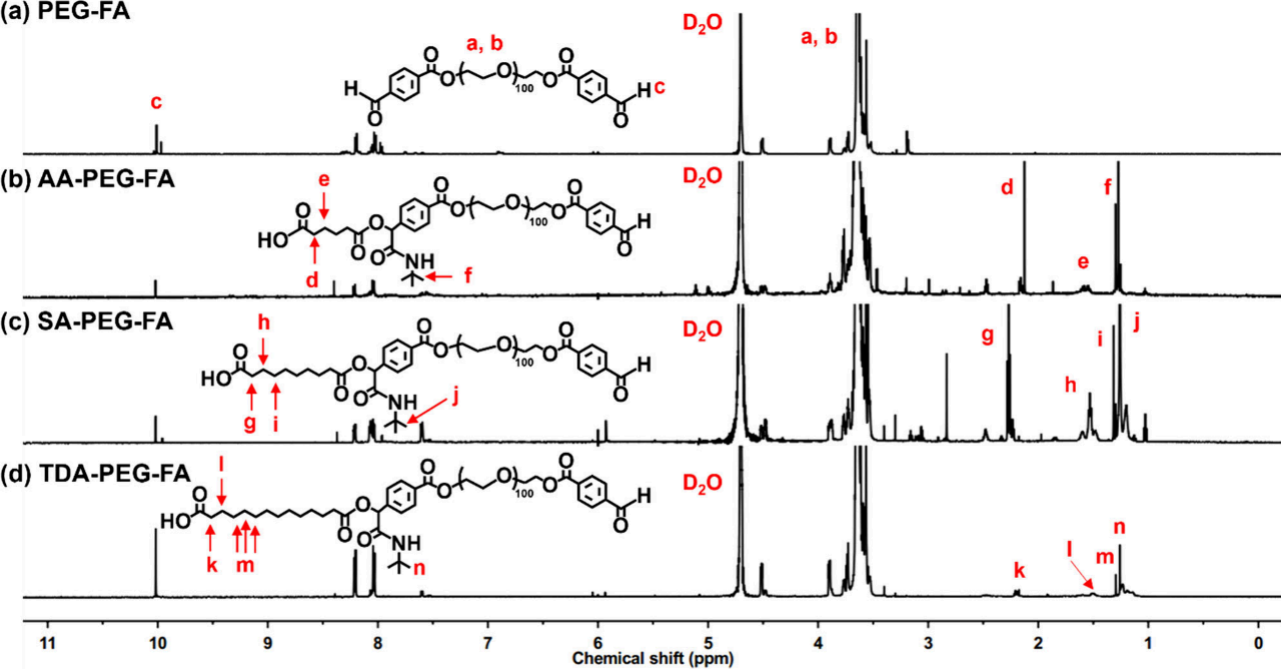
^1^H NMR spectra of (a) PEG-FA, (b) AA-PEG-FA, (c) SA-PEG-FA, and (d) TDA-PEG-FA.

In the ^1^H NMR spectrum of adipic acid-modified PEG-FA (AA-PEG-FA) (Figure 1b, Figure S5), the peak at 1.27 ppm (peak f) corresponded to the isopropyl protons (−C­(CH_3_)_3_) of tert-butyl isocyanide, and the peaks at 1.56 ppm (peak e) and 2.13 ppm (peak d) corresponded to the methylene protons (−COCH_2_CH_2_−) of adipic acid. In the ^1^H NMR spectrum of sebacic acid-modified PEG-FA (SA-PEG-FA) (Figure 1c, Figure S6), the peak at 1.27 ppm (peak j) was the signal of the isopropyl group (−C­(CH_3_)_3_) of tert-butyl isocyanide. The peaks at 1.34 ppm (peak (i), 1.52 ppm (peak h), and 2.28 ppm (peak g) corresponded to the methylene protons (−COCH_2_CH_2_CH_2_−) of sebacic acid. In the ^1^H NMR spectrum of tetradecanedioic acid-modified PEG-FA (TDA-PEG-FA) (Figure 1d, Figure S7), the peak at 1.27 ppm (peak n) represented the signal of the isopropyl group (−(CH_3_)_3_) of tert-butyl isocyanide. The peaks at 1.32 ppm (peak m), 1.48 ppm (peak l), and 2.18 ppm (peak k) corresponded to the methylene protons (−COCH_2_CH_2_(CH_2_)_3_−) of tetradecanedioic acid. The rest aldehyde amount of the dicarboxylic acid-incorporated PEG-FA derivatives was determined by NMR spectroscopy, showing the aldehyde amount of AA-PEG-FA, SA-PEG-FA, and TDA-PEG-FA were 0.28, 0.22, and 0.25 mmol/g, respectively.

In the FTIR spectrum of PEG-FA (Figure S8), the peak at 1700 cm^–1^ corresponded to the C=O stretch of the aldehyde group in the formylbenzoic acid structure, the peak at 1110 cm^–1^ corresponded to the C–O–C stretch of the ester group in the PEG structure, and the peaks at 1618 and 1200 cm^–1^ were recognized as the stretch of C≡N and C–N, respectively. The FTIR peaks mentioned above can all be observed in the spectra of AA-PEG-FA, SA-PEG-FA, and TDA-PEG-FA. The difference of dicarboxylic acid-incorporated PEG-FA derivatives in the FTIR spectra was the stretch of C=O in the dicarboxylate groups, showing the C=O peaks in AA-PEG-FA, SA-PEG-FA, and TDA-PEG-FA were 1667, 1700, and 1680 cm^–1^, respectively. The molecular weights of the synthesized macromers were determined by gel permeation chromatography (GPC), showing the weight-average molecular weight (*M*_w_) of PEG-FA, AA-PEG-FA, SA-PEG-FA, and TDA-PEG-FA was 4386 (polydispersity index (PDI) = 1.05), 4470 (PDI = 1.07), 4587 (PDI = 1.01), and 4596 (PDI = 1.03) Da, respectively (Figure S9).

### 3.2 Formations and Structural Characterizations of Hydrogels

PDH can be cross-linked using PEG-FA or its derivatives (i.e., AA-PEG-FA, SA-PEG-FA, or TDA-PEG-FA) to construct hydrogel networks through hydrazone bonds, forming PDH/PEG-FA, PDH/AA-PEG-FA, PDH/SA-PEG-FA, and PDH/TDA-PEG-FA hydrogels. Similarly, QCS cross-linked with PEG-FA or its derivatives through imine bonds can construct QCS/PEG-FA, QCS/AA-PEG-FA, QCS/SA-PEG-FA, and QCS/TDA-PEG-FA hydrogels. For hybrid hydrogel formation, PDH and QCS were mixed in different ratios according to the amounts of -NHNH2 and -NH2 present in PDH and QCS, respectively. The PDH/QCS hybrid hydrogels with -NHNH_2_: -NH_2_ ratios of 1:1, 1:2, and 1:3 were defined as P1Q1, P1Q2, and P1Q3 hydrogels, respectively. A constant amount of PEG-FA or PEG-FA derivatives was added to the PDH/QCS mixture to fabricate hybrid hydrogels. For example, P1Q1/PEG-FA, P1Q1/AA-PEG-FA, P1Q1/SA-PEG-FA, P1Q1/TDA-PEG-FA hydrogels can be obtained by adding PEG-FA or PEG-FA derivatives to the hybrid P1Q1 network.

The microstructures of hydrogels can be observed through the complementary approaches of scanning electron microscopy (SEM) and microcomputed tomography (micro-CT). SEM revealed microscopic morphology, porous structure, and surface features of hydrogels. On the other hand, micro-CT enables nondestructive three-dimensional reconstruction, showing the analysis of internal pore distribution and volumetric changes in hydrogels. Generally, SEM offers detailed surface characterization, while micro-CT provides comprehensive insights into the overall internal architecture.

The porous microstructure of the lyophilized hydrogels can be seen from the SEM images **(** Figure 2a**)**. The pore sizes of the lyophilized hydrogels were dependent on both the bonding types and macromeric cross-linkers in the network. For example, in the hydrazone-connected PDH-based hydrogels, PDH/PEG-FA, PDH/AA-PEG-FA, PDH/SA-PEG-FA, and PDH/TDA-PEG-FA hydrogels presented the average pore sizes of ∼77.4, 80.2, 79.6, and 59.4 μm, respectively **(** Figure 2b**)**. On the other hand, in the PEG-FA-cross-linked hydrogels, PDH/PEG-FA, QCS/PEG-FA, P1Q1/PEG-FA, P1Q2/PEG-FA, and P1Q3/PEG-FA hydrogels presented the average pore sizes of ∼77.4, 121.4, 78.0, 80.1, and 84.0 μm, respectively (Figure 2b). Among the 20 hydrogels, QCS/AA-PEG-FA hydrogel presented the largest pore size of ∼ 144.4 μm, and PDH/TDA-PEG-FA hydrogel possessed the smallest pore size of ∼59.4 μm (Figure 2b).

**Figure 2:**
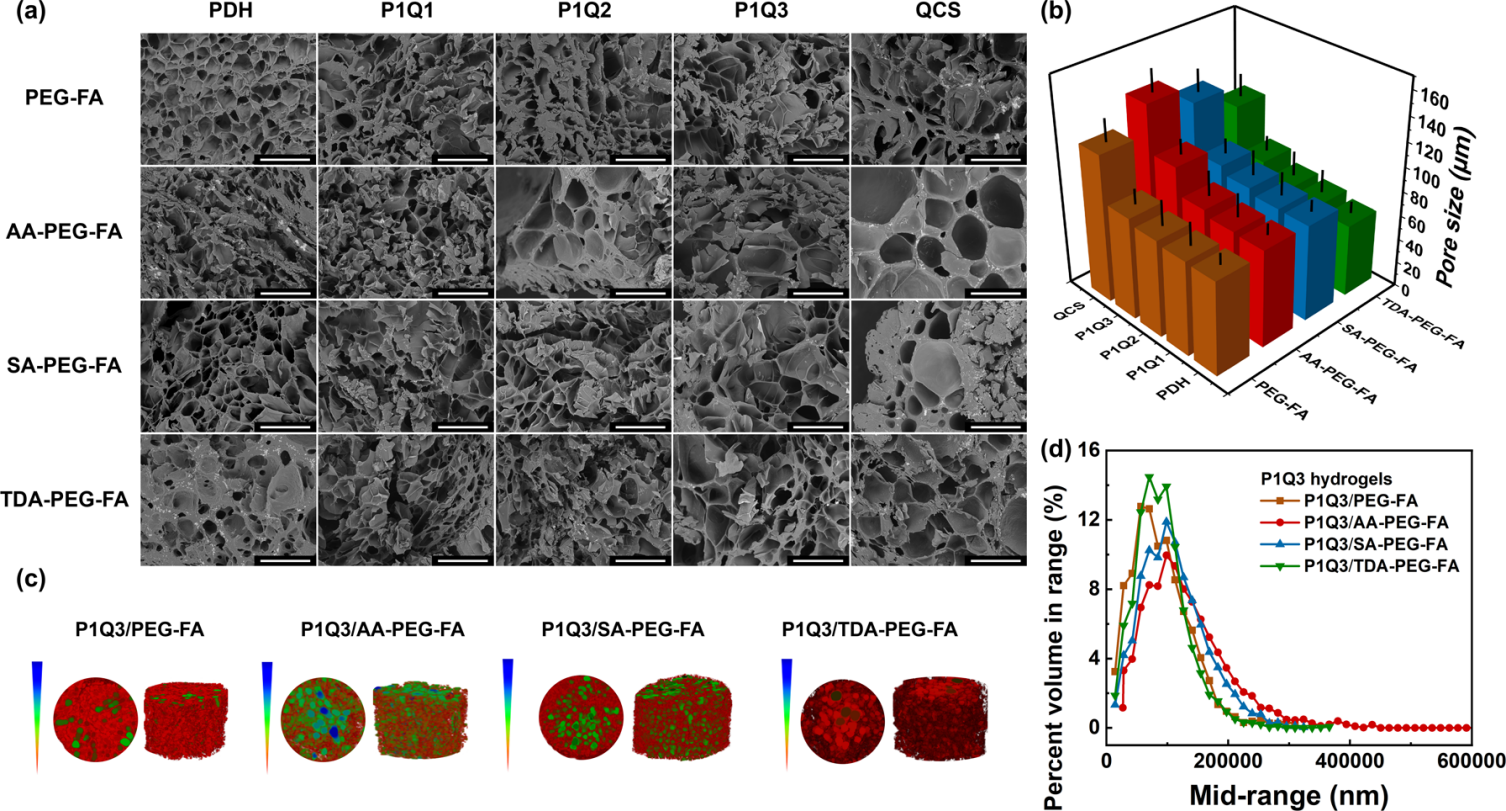
(a) Representative SEM images of hydrogels. Scale bar: 300 μm. (b) Pore sizes of hydrogels analyzed by SEM (c) micro-CT images of hydrogels, where the blue blocks were the parts with larger pores, the green blocks were the parts with medium pores, and the red blocks were the regions with smaller pores. (d) Pore size distribution of hydrogels analyzed by micro-CT.

The P1Q3-based hydrogels were further analyzed using micro-CT. The micro-CT images showed that the P1Q3/PEG-FA and P1Q3/TDA-PEG-FA hydrogels exhibited more red regions, indicative of smaller pore sizes, compared to the P1Q3/AA-PEG-FA and P1Q3/SA-PEG-FA hydrogels (Figure 2c). The average pore sizes of P1Q3/PEG-FA, P1Q3/AA-PEG-FA, P1Q3/SA-PEG-FA, and P1Q3/TDA-PEG-FA hydrogels were 90.8, 142.3, 110.2, and 90.5 μm, respectively (Figure 2d, Table S1). Open porosity in micro-CT analysis refers to interconnected pores facilitating fluid transport, while closed porosity refers to isolated pores without direct pathways to the external environment. The open porosity of P1Q3/PEG-FA, P1Q3/AA-PEG-FA, P1Q3/SA-PEG-FA, and P1Q3/TDA-PEG-FA hydrogels were 40.5%, 70.7%, 69.1%, and 64.0%, respectively. The closed porosity of P1Q3/PEG-FA, P1Q3/AA-PEG-FA, P1Q3/SA-PEG-FA, and P1Q3/TDA-PEG-FA hydrogels were 1.6%, 0.5%, 0.1%, and 0.6%, respectively. The total porosity was represented as the volume fraction of the air. The total porosity of P1Q3/PEG-FA, P1Q3/AA-PEG-FA, P1Q3/SA-PEG-FA, and P1Q3/TDA-PEG-FA hydrogels were 41.4%, 70.8%, 69.2%, and 64.3%, respectively.

### 3.3 Compression, Tensile, and Adhesion Tests of Hydrogels

The mechanical properties of hydrogels were determined through compression tests, where the 10–20% strain region in the stress–strain curves of hydrogels was used to calculate the compression modulus of the hydrogels (Figure S10). Taking PDH-based hydrogels as examples, PDH/PEG-FA, PDH/AA-PEG-FA, PDH/SA-PEG-FA, and PDH/TDA-PEG-FA hydrogels exhibited compression moduli of ∼17.5, 9.2, 14.2, and 21.1 kPa, respectively (Figure 3a). On the other hand, PEG-FA-cross-linked hydrogels, including PDH/PEG-FA, QCS/PEG-FA, P1Q1/PEG-FA, P1Q2/PEG-FA, and P1Q3/PEG-FA, showed compression moduli of ∼17.5, 4.1, 14.2, 8.6, and 7.2 kPa, respectively (Figure 3a). Among the 20 hydrogels analyzed, PDH/TDA-PEG-FA hydrogel demonstrated the highest compression modulus of ∼21.1 kPa, while the QCS/AA-PEG-FA hydrogel had the lowest compression modulus of ∼1.4 kPa (Figure 3a).

**Figure 3:**
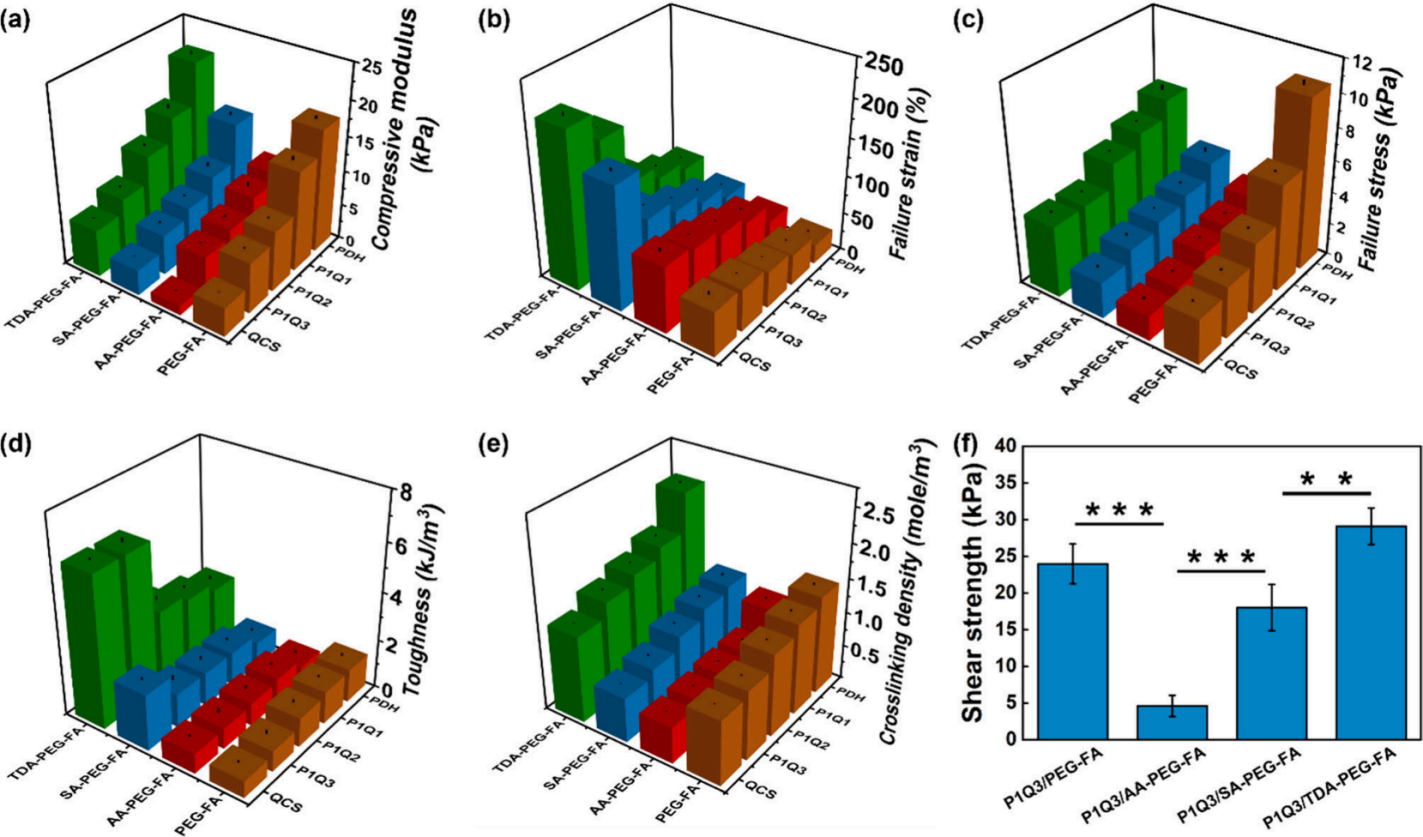
(a) Compressive modulus, (b) failure strain, (c) failure stress, (d) toughness, and (e) cross-linking density of hydrogels. (f) Shear strength of P1Q3-based hydrogels in adhesion tests. Significance was set as *p* < 0.05 with ** or *** indicating *p* < 0.01 or 0.001, respectively.

The cyclic compression test was further used to observe the fatigue resistance of hydrogels, and the test was performed to a maximum of 50% strain in 5 cycles (Figure S11a–d). Hysteresis ratio, the ratio of the hysteresis loop area to the total integrated area under the loading curve, was further calculated for each hydrogel.^24^ The hysteresis ratio of P1Q3/PEG-FA (0.68) was larger than other hydrogels, including P1Q3/AA-PEG-FA (0.24), P1Q3/SA-PEG-FA (0.17), and P1Q3/TDA-PEG-FA (0.15) hydrogels (Figure S11e).

The tensile test was performed to determine the failure strain, failure stress, and toughness of hydrogels. The representative stress–strain curves of the hydrogels are shown in Figure S12. For the failure strain analysis, using PDH-based hydrogels as examples, the failure strains for the PDH/PEG-FA, PDH/AA-PEG-FA, PDH/SA-PEG-FA, and PDH/TDA-PEG-FA hydrogels were ∼25.3%, 32.9%, 43.4%, and 60.3%, respectively (Figure 3b). On the other hand, the PEG-FA-cross-linked hydrogels, which included PDH/PEG-FA, QCS/PEG-FA, P1Q1/PEG-FA, P1Q2/PEG-FA, and P1Q3/PEG-FA hydrogels, exhibited failure strains of ∼25.3%, 59.2%, 40.6%, 47.7%, and 55.5%, respectively (Figure 3b). Among the 20 hydrogels, the QCS/TDA-PEG-FA hydrogel displayed the highest failure strain of ∼ 206.6%, whereas the PDH/PEG-FA hydrogel showed the lowest failure strain of ∼25.3% (Figure 3b).

In the failure stress analysis, for example, the failure stresses for the PDH/PEG-FA, PDH/AA-PEG-FA, PDH/SA-PEG-FA, and PDH/TDA-PEG-FA hydrogels were ∼10.5, 3.4, 4.6, and 7.6 kPa, respectively (Figure 3c). In addition, the PDH/PEG-FA, QCS/PEG-FA, P1Q1/PEG-FA, P1Q2/PEG-FA, and P1Q3/PEG-FA hydrogels exhibited failure stresses of ∼10.5, 2.8, 6.6, 4.4, and 3.2 kPa, respectively (Figure 3c). Among the 20 hydrogels analyzed, the PDH/PEG-FA hydrogel demonstrated the highest failure stress of ∼10.5 kPa, while the QCS/AA-PEG-FA hydrogel showed the lowest failure stress of ∼1.5 kPa (Figure 3c).

In the toughness analysis, the toughness values for the PDH/PEG-FA, PDH/AA-PEG-FA, PDH/SA-PEG-FA, and PDH/TDA-PEG-FA hydrogels were ∼1.4, 0.7, 0.9, and 2.6 kJ/m^3^, respectively (Figure 3d). Additionally, the PDH/PEG-FA, QCS/PEG-FA, P1Q1/PEG-FA, P1Q2/PEG-FA, and P1Q3/PEG-FA hydrogels exhibited toughness values of ∼1.4, 0.6, 1.3, 1.2, and 0.8 kJ/m^3^, respectively (Figure 3d). Among the 20 hydrogels evaluated, the QCS/TDA-PEG-FA hydrogel exhibited the highest toughness of ∼6.2 kJ/m^3^, while the QCS/PEG-FA hydrogel displayed the lowest toughness of ∼0.6 kJ/m^3^ (Figure 3d).

The cross-linking density of the hybrid hydrogels can be further determined by the rheological tests along with the following equation.

$$G^{\prime }=\nu RT$$

In which, *G’* (Pa) is storage modulus, ν (mol/m^3^) represents cross-linking density, R (8.314 J/K·mol) is gas constant, and T represents the temperature in 298.15 K.^25^ The oscillation time sweeps of hydrogels were shown in Figure S13, and the detailed analyses (i.e., storage modulus and cross-linking density) were presented in Figure 3e and Table S2. For example, the cross-linking densities for the PDH/PEG-FA, PDH/AA-PEG-FA, PDH/SA-PEG-FA, and PDH/TDA-PEG-FA hydrogels were ∼ 1.43, 0.99, 1.12, and 2.29 mol/m^3^, respectively (Figure 3e, Table S2). Additionally, the PDH/PEG-FA, QCS/PEG-FA, P1Q1/PEG-FA, P1Q2/PEG-FA, and P1Q3/PEG-FA hydrogels exhibited cross-linking densities of ∼1.43, 0.94, 1.32, 1.20, and 1.01 mol/m^3^, respectively (Figure 3e, Table S2). Among the 20 hydrogels evaluated, the PDH/TDA-PEG-FA hydrogel exhibited the highest cross-linking density of ∼2.29 mol/m^3^, while the QCS/AA-PEG-FA hydrogel displayed the lowest cross-linking density of ∼0.53 mol/m^3^ (Figure 3e, Table S2). The trend in cross-linking density among the hydrogels aligned with their measured compressive modulus and toughness, indicating that hydrogels with higher cross-linking densities exhibited enhanced mechanical performance.

It has been reported that QCS-containing hydrogels can adhere to tissue surfaces due to the amino and quaternary ammonium groups in QCS to allow it to interact with tissues through hydrogen bonding and electrostatic forces.^26,27^ Here, the P1Q3-based hydrogels were placed between two pieces of pig skin to examine their adhesion property through shear strength analysis. The stress–strain curves of P1Q3-based hydrogels are shown in Figure S14, and the shear strength of P1Q3/PEG-FA, P1Q3/AA-PEG-FA, P1Q3/SA-PEG-FA, and P1Q3/TDA-PEG-FA hydrogels were ∼24.0, 4.6, 18.0, and 29.1 kPa, respectively (Figure 3f). The P1Q3/TDA-PEG-FA hydrogel exhibited the strongest adhesion performance, which can be attributed to its high tensile strength and toughness.^28^

### 3.4 Water Content and Stability of Hydrogels

The water content and degradation ability of hydrogels are highly related to their biomedical applications. In general, a high water content of hydrogels enhances their biocompatibility,^29^ while excessive water content can accelerate the degradation of the hydrogel structure and hinder its performance in biomedical use.^30^ Here, the water content of the hydrogels was measured by soaking lyophilized hydrogels in phosphate-buffered saline (PBS) for 12 h and weighing the hydrogels at specific time intervals. For example, the water contents of the PDH/PEG-FA, PDH/AA-PEG-FA, PDH/SA-PEG-FA, and PDH/TDA-PEG-FA hydrogels were ∼ 91.9%, 91.0%, 90.8%, and 87.2%, respectively (Figure S15a). Besides, the PDH/PEG-FA, QCS/PEG-FA, P1Q1/PEG-FA, P1Q2/PEG-FA, and P1Q3/PEG-FA hydrogels exhibited water content of ∼ 91.9%, 96.4%, 92.2%, 92.3%, and 93.4%, respectively (Figure 4a). Among the 20 hydrogels tested, the QCS/PEG-FA hydrogel exhibited the highest water content of ∼96.4%, while the PDH/TDA-PEG-FA hydrogel displayed the lowest water content of ∼87.2% (Figure S15).

**Figure 4:**
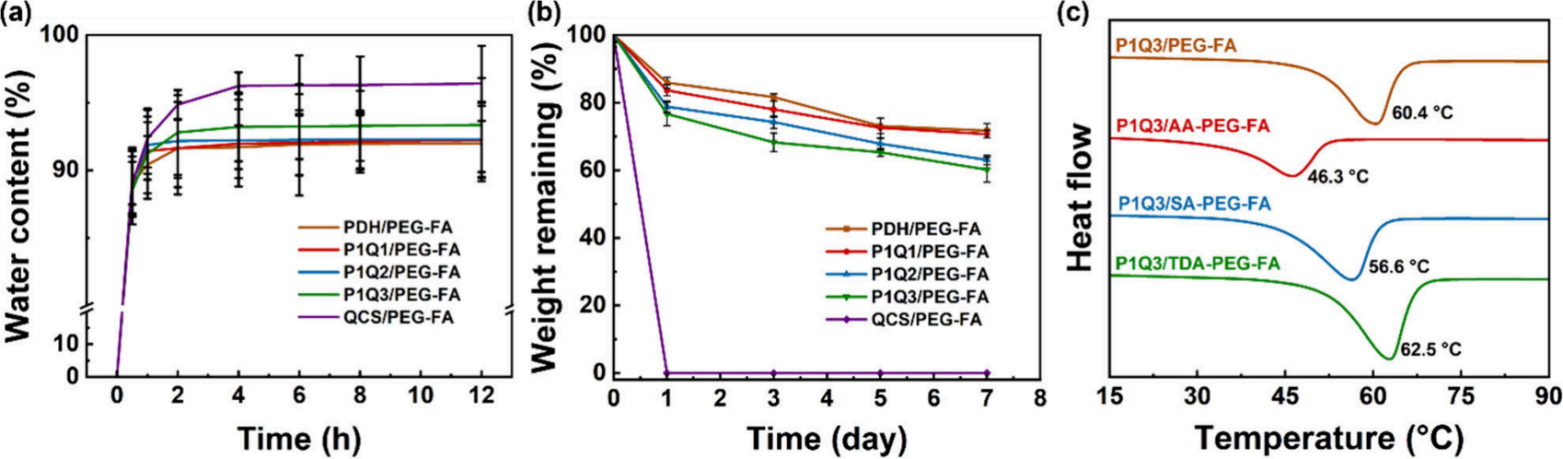
(a) Water contents and (b) weight remaining of hydrogels. (c) DSC traces and *T*_m_ values of P1Q3-based hydrogels.

The degradation of hydrogels was assessed by immersing the lyophilized hydrogels in PBS solution at 37 °C for 7 days to evaluate their stability in a physiological environment. For example, the remaining weights of the PDH/PEG-FA, PDH/AA-PEG-FA, PDH/SA-PEG-FA, and PDH/TDA-PEG-FA hydrogels were ∼71.7%, 68.9%, 70.9%, and 75.3%, respectively (Figure S16a). Additionally, the PDH/PEG-FA, QCS/PEG-FA, P1Q1/PEG-FA, P1Q2/PEG-FA, and P1Q3/PEG-FA hydrogels showed remaining weights of ∼71.7%, 0%, 70.7%, 63.0%, and 60.2%, respectively (Figure 4b). Among the 20 hydrogels, the PDH/TDA-PEG-FA hydrogel had the highest remaining weight of 75.33%, while the QCS-based hydrogel exhibited the lowest remaining weight and almost completely degraded (Figure S16). Additionally, the morphological changes of the P1Q3-based hydrogel structure after immersion were further investigated using SEM. It was noticed that the pore sizes of the hydrogels became larger after long-term immersion (Figure S17a). P1Q3/PEG-FA, P1Q3/AA-PEG-FA, P1Q3/SA-PEG-FA, and P1Q3/TDA-PEG-FA hydrogels presented the average pore sizes of ∼118.2, 198.2, 179.3, and 110.2 μm, respectively, after 7 days of immersion (Figure S17b). The remaining weights of the P1Q3/PEG-FA, P1Q3/AA-PEG-FA, P1Q3/SA-PEG-FA, and P1Q3/TDA-PEG-FA hydrogels were ∼60.2%, 52.4%, 57.5%, and 63.3%, respectively, after 7 days of immersion (Figure S16e). Therefore, the SEM observations aligned with the weight retention analysis of the hydrogels, revealing that prolonged immersion led to greater polymer loss and the formation of larger pores within the network.

The thermal stability of the hydrogels was revealed by determining their melting temperature (*T*_m_) through differential scanning calorimetry (DSC).^31^ The *T*_m_ values of P1Q3/PEG-FA, P1Q3/AA-PEG-FA, P1Q3/SA-PEG-FA, and P1Q3/TDA-PEG-FA hydrogels were 60.4, 46.3, 56.6, and 62.5 °C, respectively (Figure 4c), indicating that P1Q3/TDA-PEG-FA hydrogel exhibited the highest thermal stability due to its dense networks.^32^

### 3.5 Motion Monitoring and Biocompatibility of Hydrogels

Elastic hydrogels with conductivity have been applied in wearable devices for motion monitoring.^33^ The mechanical properties of hydrogels in wearable devices play a crucial role in ensuring the stability of motion detection. Also, an ideal hydrogel should possess both excellent stretchability and a stable structure to prevent rapid degradation. To meet the requirements of wearable devices, P1Q3-based hydrogels were selected as representative examples to demonstrate their potential for motion monitoring.

The conductivity of the P1Q3-based hydrogels could be attributed to the amino and quaternary ammonium groups of QCS that facilitate charge transport in the hydrogel network.^34^ Electrochemical impedance spectroscopy (EIS) was used to quantify the conductivity of the P1Q3-based hydrogel, with the EIS probes attached to both ends of the hydrogel. The conductivities of P1Q3/PEG-FA, P1Q3/AA-PEG-FA, P1Q3/SA-PEG-FA, and P1Q3/TDA-PEG-FA hydrogels were 0.16, 0.17, 0.17, and 0.15 S m^–1^, respectively (Figure 5a). On the other hand, gauge factor (GF), a coefficient obtained by combining a materials testing system and a multimeter, is an important indicator to evaluate the strain sensitivity of hydrogels determined by the slope of the ΔR/R_0_ curve under different strains, where ΔR represents the change in electrical resistance of hydrogels during deformation and R_0_ is the initial resistance of the hydrogel. When the hydrogel was stretched by the machine, the conductive pathways became narrow, which increased resistance. A greater increase in resistance indicates higher strain sensitivity of the hydrogel.^35^ The GF of P1Q3/PEG-FA, P1Q3/AA-PEG-FA, P1Q3/SA-PEG-FA, and P1Q3/TDA-PEG-FA hydrogels were 1.12, 0.05, 0.06, and 1.29, respectively (Figure 5b). The P1Q3/TDA-PEG-FA hydrogel showed the best sensitivity over P1Q3-based hydrogels due to its smallest pore size and highest compression modulus among the P1Q3-based hydrogels. The dense and stable structure of P1Q3/TDA-PEG-FA hydrogel restricted ion mobility to cause a slight decrease in conductivity and increased sensitivity to changes in resistance.^36^

**Figure 5:**
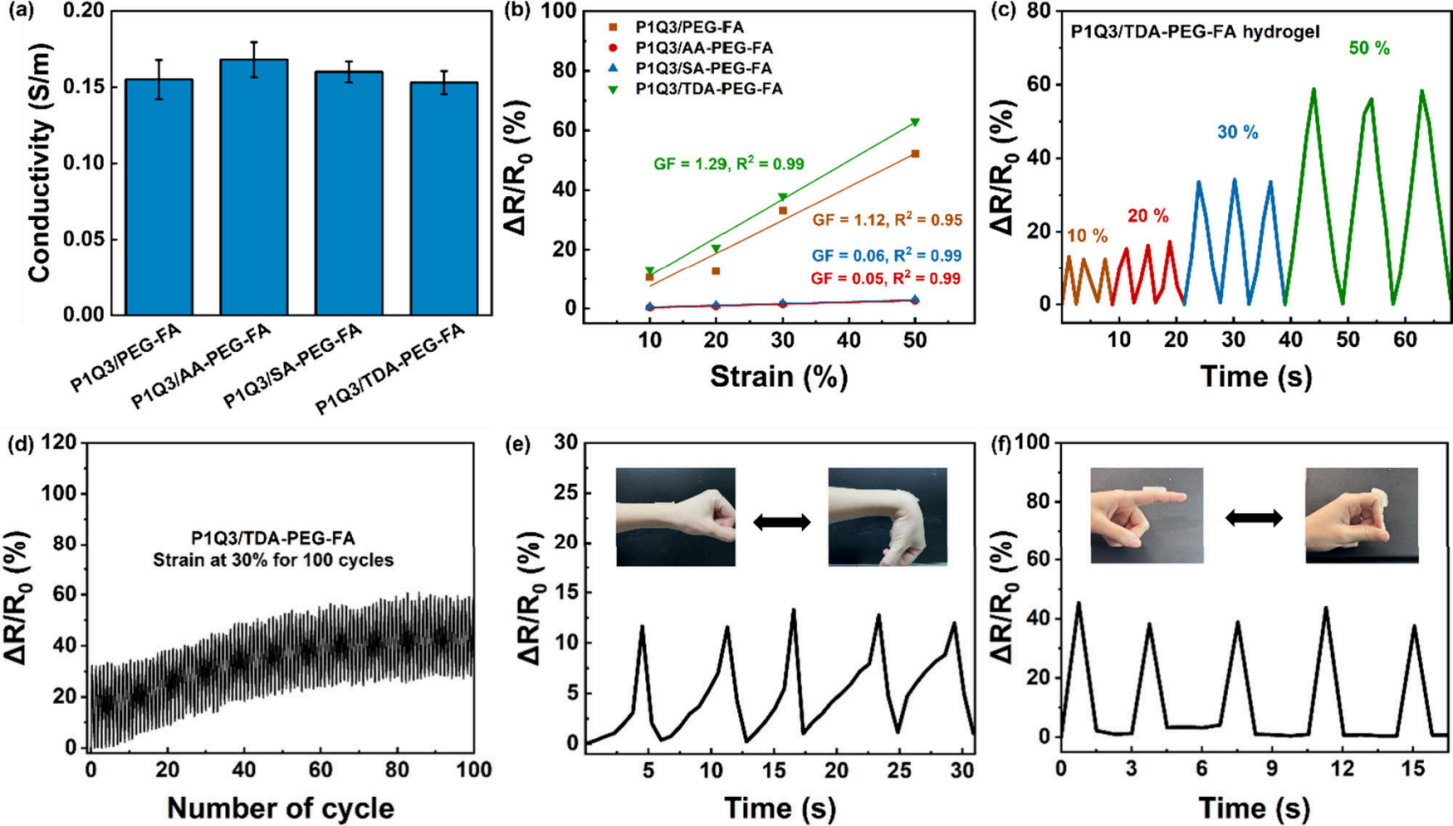
Electrical properties measurement. (a) Conductivity of P1Q3-based hydrogels. (b) Gauge factor of P1Q3-based hydrogels. (c) Relative resistance changes of P1Q3/TDA-PEG-FA hydrogel under different strains. (d) Cyclic stability of P1Q3/TDA-PEG-FA hydrogel for 100 cycles between 0 and 30% strain. Motion detection of (e) wrist joint and (f) finger joint using P1Q3/TDA-PEG-FA hydrogel.

Cyclic tensile test was applied to different strains of P1Q3/TDA-PEG-FA hydrogel over 3 cycles, and the distinct relative resistance changes under different deformations were observed (Figure 5c). Furthermore, the relative resistance changes were reproducible, showing that the resistance change can be recovered during cyclic extending to 30% strain for 100 cycles (Figure 5d). It was also observed that the resistance variation increased slightly during the 100 cycles, which was attributed to gradual water loss in the sample during the loading process and caused the decrease in conductivity and a subsequent increase in resistance.^37^ To further demonstrate the potential of P1Q3/TDA-PEG-FA hydrogel in wearable devices, we demonstrated the strong adhesive performance of the P1Q3/TDA-PEG-FA hydrogel on a finger joint, where it remained securely attached and did not detach during flexion and movement, indicating robust adhesion under dynamic conditions (Figure S18). Furthermore, P1Q3/TDA-PEG-FA hydrogel was placed on the person’s wrist and finger joints. Once the person consistently bent the tested part from 0° to 90°, the relative resistance changes in the movement of the wrist (Figure 5e) and finger joints (Figure 5f) both showed reproducibility and stability. It was noticed that the resistance change was more significant when the hydrogel was placed on the finger joint than the wrist joint, possibly due to the wrist being bent less (smaller deformation) than the finger when moving. Therefore, the resistance change of the wrist joint was smaller than the finger joint.^38^ The strain-responsive behavior of the hydrogels during electrical measurements was further examined using the P1Q3/TDA-PEG-FA hydrogel applied directly to the finger joint to monitor finger bending motions (Figure S19). When the finger remained in a neutral position (0° bend), the electrical resistance of the hydrogel remained stable with no significant variation. Upon bending the finger to 45° and 90°, the hydrogel underwent about 50% and 100% elongation relative to its original length, respectively. Correspondingly, the resistance changes at 90° were more pronounced than those observed at 45°, indicating a clear correlation between the extent of hydrogel elongation and the change in electrical resistance.

The *in vitro* cytocompatibility of hydrogels was evaluated through the alamarBlue and live/dead staining assays. The P1Q3-based hydrogels were soaked in the serum-containing media for 24 h to prepare the extract solutions for cellular studies. Mouse embryonic fibroblast (MEF) cells cultured with the extract solutions of the hydrogels continued to proliferate over 3 days (Figure S20), showing the biocompatibility of these hydrogels for applications in biomedicine and wearable devices.

## 4 Discussion

In this study, two functionalized polymers (i.e., PDH and QCS) were cross-linked with the aldehyde-modified PEG-based macromers (i.e., PEG-FA, AA-PEG-FA, SA-PEG-FA, and TDA-PEG-FA) to construct hydrogels through dynamic cross-links. PDH, featuring hydrazine (NHNH_2_) groups, formed hydrazone bonds with aldehyde-containing macromers. In contrast, QCS, with amine (NH_2_) groups, reacted with aldehyde-containing macromers through imine bonds. It has been reported that the delocalization of hydrazone electrons, facilitated by the electronegativity of the hydrazide carbonyl oxygen, results in greater bond stabilization of hydrazone bonds than imine bonds.^39^ Therefore, the superior stability of hydrazone bonds compared to imine bonds affected the structures and properties of PDH-based, QCS-based, and PDH/QCS hybrid hydrogels. PDH-based hydrogels generally exhibited smaller pore sizes, greater cross-linking density and mechanical properties (i.e., compression modulus, failure stress, and toughness), as well as enhanced stability compared to QCS-based hydrogels, which were softer and exhibited better elongation and flexibility (Scheme 3a). Particularly, there was an obvious difference between the hydrazone-cross-linked and the imine-cross-linked networks in the degradation tests of hydrogels. The weight of PDH/PEG-FA hydrogel remained at ∼71% after 7 days of immersion in PBS at 37 °C, while the QCS/PEG-FA hydrogel was completely degraded after 1 day. Upon mixing the PDH and QCS polymers, the structures and properties of the hybrid hydrogels can be fine-tuned by adjusting the NH_2_:NHNH_2_ ratios in the networks. In general, P1Q1-based hydrogels demonstrated superior mechanics and stability but lower extensibility than P1Q3-based hydrogels. The reduced failure strain in P1Q1-based hydrogels was due to the stability of the hydrazone bonds that limited the extensibility of the hydrogel network. A radar chart was plotted as an example to compare P1Q1/PEG-FA and P1Q3/PEG-FA hydrogels (Scheme 3b). Overall, raising the hydrazone/imine ratio in the network increased the mechanical properties and stability of the PDH/QCS hybrid hydrogels, owing to the inherent stability of hydrazone bonds. The bonding types in the network influenced the structures and properties of hydrogels, highlighting the balance between mechanical strength and flexibility in hybrid hydrogels and the complementary roles of hydrazone and imine bonds in modulating their characteristics and performance.

**Scheme 3:**
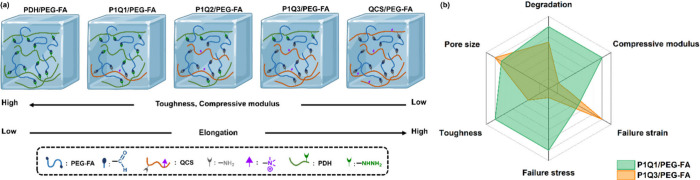
(a) Five Types of Hydrogels Cross-Linked with PEG-FA for the Comparison of Their Mechanical Properties and (b) Radar Plot for the Properties of P1Q1/PEG-FA and P1Q3/PEG-FA Hydrogels

On the other hand, the hydrophobic segment lengths in the macromeric cross-linkers also determined the performance of hydrogels. Our findings revealed that the P1Q3/TDA-PEG-FA hydrogel, with the longest hydrophobic segments in the macromeric cross-linkers, exhibited an elongation of up to 170.6% and excellent antifatigue performance as well as high stability in PBS. A radar chart was plotted for the P1Q3-based hydrogels to clearly describe the improved properties of hydrogels by adding hydrophobic segments in the macromeric cross-linkers (Scheme 4a). Overall, the longer the hydrophobic segments of macromeric cross-linkers in the hydrogels, the greater the cross-linking density, strength, ductility, toughness, fatigue resistance, and stability of the hydrogels.

**Scheme 4:**
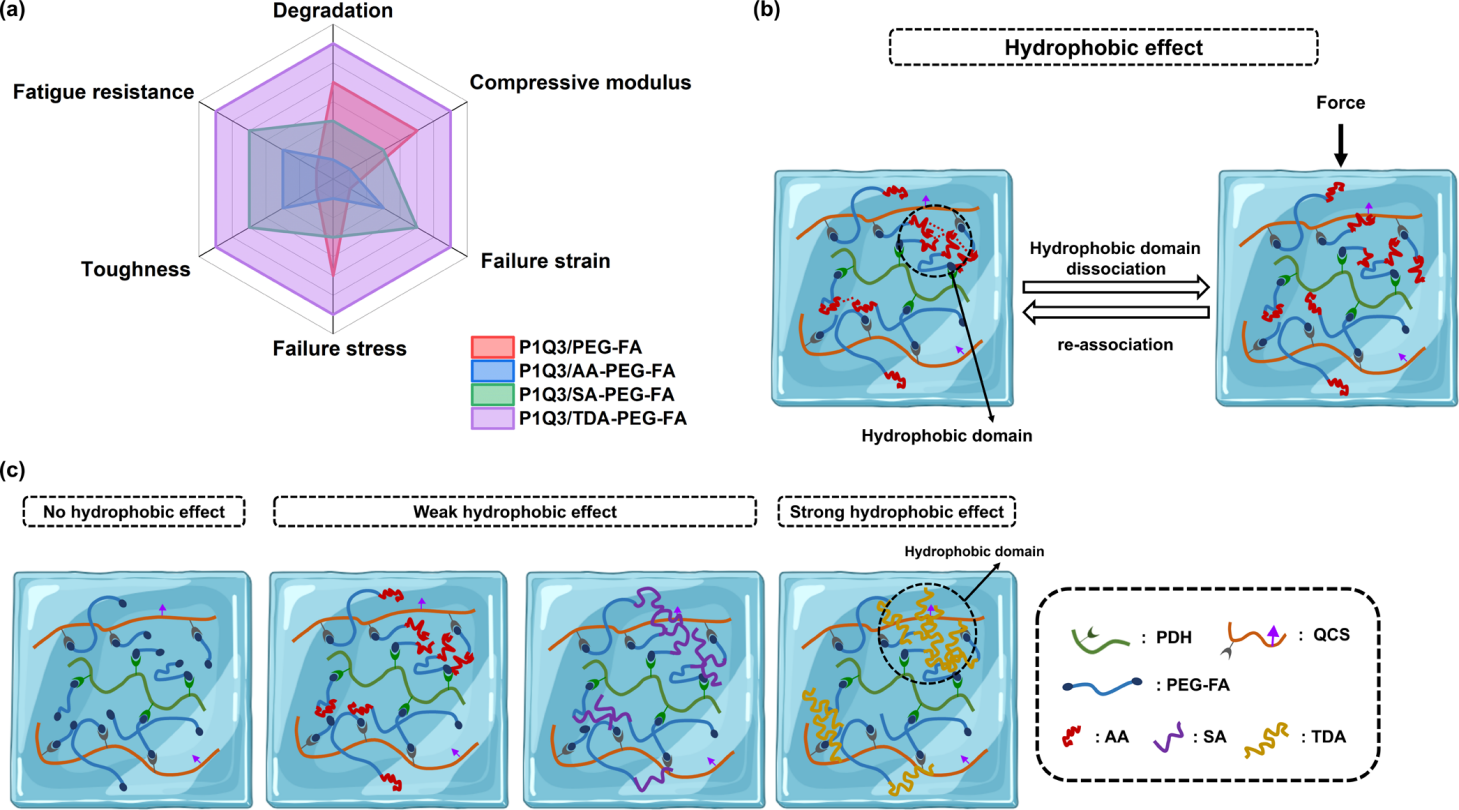
**(**a) Radar Plot for the Properties of P1Q3-Based Hydrogels, (b) Hydrophobic Effect in Hydrogels, and (c) Hydrophobic Effect of PEG-FA, AA-PEG-FA, SA-PEG-FA, and TDA-PEG-FA in Hydrogel Networks

Recent literature has highlighted the role of the hydrophobic domains in the hydrogels. For example, Gao et al. utilized poly­(butyl acrylate) (PBA) latex particles as hydrophobic cross-linkers, incorporating alkyl chains to enhance their properties, where the hydrogel achieved an excellent failure stress of 1.2 MPa and a failure strain of 2336%.^40^ Xu et al. formed hydrogels through acrylamide in free radical polymerization and reinforced them with sodium dodecyl sulfate (SDS) and dodecyl dimethyl betaine (BS-12), which brought hydrophobic interactions and physical cross-linking.^41^ The hydrogels achieved a high tensile strength of 700 kPa as the hydrophobic segments can self-assemble to serve as cross-linking centers to enhance the mechanical strength of the hydrogel network.^42^ This mechanical strength enhancement is achieved through microphase separation driven by hydrophobic interactions. Generally, hydrophobic associations are a unique feature of amphiphilic molecules containing both hydrophilic and hydrophobic segments (Scheme 4b). In aqueous environments (like hydrogels), these associations are entropy-driven processes that minimize unfavorable interactions between hydrophobic segments and water. The equilibrium between hydrophobic domains is dynamic; by applying external forces, reversible dissociation dissipates energy within the hydrogel matrix. The hydrophobic segments within the hydrogels aggregate due to hydrophobic forces, behaving like springs that resist rupture by distributing external stresses. Therefore, tuning the contrast between hydrophobic and hydrophilic segments, as well as the type and size of the hydrophobic domains, can modulate the mechanical properties of the hydrogel.

In this study, the aldehyde-containing PEG-based macromers cross-linked the polymeric networks via hydrazone and/or imine bonds. Hydrogels cross-linked with PEG-FA exhibited stable networks, attributed to the free aldehyde groups at both ends of PEG-FA. For instance, the P1Q3/PEG-FA hydrogel demonstrated excellent compressive modulus and extensibility, as well as showing significant hysteresis during cyclic compression tests. However, it was noticed that the failure strain of the QCS/PEG-FA hydrogel was only ∼59%, which was significantly lower than that of hydrogels cross-linked with macromers containing hydrophobic segments (failure strain> 86.9%). The length of hydrophobic chain segments in aldehyde-containing PEG-FA derivatives significantly influenced the structures and mechanics of hydrogels. Specifically, PDH/QCS-based hydrogels cross-linked with TDA-PEG-FA, which has a longer hydrophobic segment, exhibited smaller pore sizes, higher cross-linking density, enhanced mechanical properties (i.e., compression modulus, failure stress, and toughness), and superior stability compared to those cross-linked with AA-PEG-FA or SA-PEG-FA. These longer hydrophobic segments also reduced the hysteresis effect in the hydrogel network, with hydrogels cross-linked with TDA-PEG-FA demonstrating the best antifatigue performance. These observations can be due to the hydrophobic domains acting as physical cross-links in the network, enhancing the mechanical strength, toughness, and resilience of hydrogels (Scheme 4c). Shorter hydrophobic segments in the macromers create smaller, less interconnected domains, resulting in weaker hydrophobic interactions and lower mechanical integrity, leading to greater susceptibility to fatigue. On the other hand, longer hydrophobic chains in the macromers promote stronger intermolecular interactions, yielding improved mechanical properties to withstand higher stress and elongate during stretching.

The incorporation of hydrophobic domains within hydrogel networks to modulate their properties has been previously demonstrated using hydrophobic nanoparticles^40,43^ or copolymers containing hydrophobic segments.^44,45^ In this work, we developed PEG-based macromeric cross-linkers with systematically varied hydrophobic segment lengths to reveal the role of the structural differences in affecting the structures and properties of dextran/chitosan hybrid hydrogels.

To facilitate a clear comparison with existing dextran/chitosan hybrid hydrogels, we have compiled Table 1 that systematically outlines the functional groups present in the polymers, along with the cross-linkers and/or cross-linking chemistries employed within the networks. In most reported dextran/chitosan hybrid hydrogels, dextran is typically oxidized to introduce aldehyde groups, enabling the formation of dynamic imine cross-links with the amino groups of chitosan. Some systems utilize additional cross-linkers, while these are often integrated into the network through noncovalent interactions such as electrostatic interactions or hydrogen bonding. In this work, our approach involved the modification of dextran with hydrazide groups rather than aldehydes. The hydrazide-functionalized dextran was mixed with quaternized chitosan in varying ratios, allowing for the formation of both hydrazone and imine bonds in the presence of aldehyde-functionalized macromeric cross-linkers. Adjusting the polymer mixing ratio and tuning the structure of the cross-linker provides a versatile and efficient means of modulating the properties of dextran/chitosan hydrogels without the need for extensive structural modifications of the base polymers. Notably, our system incorporates both hydrophobic associations and hydrazone linkages, distinguishing it from current dextran/chitosan systems and broadening the potential applications and functional diversity of these hydrogels.

**Table 1:** Dextran/Chitosan Hybrid Hydrogels

Functional groups on dextran	Functional groups on chitosan	Cross-linker	Cross-linking chemistry	Reference
Aldehyde	Amine, carboxyethyl group	None	Imine bond, hydrogen bond	^46^
Aldehyde	Amine, pyrogallol group	None	Imine bond, hydrogen bond	^11^
Aldehyde	Amine, hydroxypropyl group	None	Imine bond, hydrogen bond	^47^
Aldehyde	Amine	None	Imine bond, hydrogen bond	^48^
Aldehyde	Amine, choline phosphate group	None	Imine bond, hydrogen bond, electrostatic forces	^49^
Aldehyde	Amine, quaternary ammonium group	Tetrasulfide bond-bridged mesoporous silica nanoparticles	Imine bond, hydrogen bond, electrostatic forces	^50^
Aldehyde	Amine, quaternary ammonium group	Cuttlefish ink-based melanin	Imine bond, hydrogen bond	^51^
Aldehyde	Amine, carboxyl group	Tannic acid	Imine bond, hydrogen bond, Michael’s type bond	^52^
Hydrazide	Amine, quaternary ammonium group	Dicarboxylic acid-incorporated PEG-FA (aldehyde contained)	Imine bond, hydrazone bond, hydrogen bond, electrostatic forces, hydrophobic interaction	This work

The dynamically cross-linked network of the PDH/QCS hybrid hydrogels possesses shear-thinning and self-healing properties, making it well-suited for 3D printing^53,54^ to create complex and customized architectures. To further enhance the long-term stability of these hydrogels, a third cross-linking mechanism^55,56^ or the incorporation of nanomaterial-based fillers^55,57^ could be introduced to reinforce the structural integrity of the network. Additionally, the inherent mechanical sensing capabilities of the PDH/QCS hydrogels can be integrated with chemical or optical sensing elements, enabling the development of multimodal sensing platforms.

As a conductive hydrogel system, the electrical performance of the PDH/QCS hybrid hydrogels can be further enhanced by incorporating intrinsically conductive polymers (e.g., polyaniline^58^ or polypyrrole^59^). This integration would optimize the conductivity pathways within the network and improve the sensitivity and responsiveness in mechanical sensing applications. Moreover, to ensure reliable performance under extreme environmental conditions, future designs should focus on imparting antifreezing, antidrying, and antiswelling properties, broadening the operational range and application scenarios of these hydrogels.^60^ Incorporating bioactive molecules (e.g., growth factors,^61^ proteins,^62^ and functional peptides^63^) could further enhance biocompatibility and biofunctionality, promoting improved hydrogel-tissue interactions. These advancements would significantly strengthen the potential of conductive PDH/QCS hybrid hydrogels for use in diverse biomedical applications, including tissue engineering, bioelectronics, and implantable sensors.

## 5 Conclusion

In summary, we have developed 20 types of hydrogels using various polymeric components (i.e., PDH, QCS, P1Q1, P1Q2, and P1Q3) and macromeric cross-linkers (i.e., PEG-FA, AA-PEG-FA, SA-PEG-FA, and TDA-PEG-FA) through dynamic bonding. Notably, P1Q3/TDA-PEG-FA hydrogel demonstrated exceptional stretchability (170.6%), high toughness (2.6 kJ/m^3^), and remarkable stability among the tested hydrogels. This study revealed that carefully adjusting the hydrazone/imine ratio in the network and the hydrophobic segment lengths of the macromeric cross-linkers effectively balanced porous microstructures, cross-linking density, mechanical strength, stretchability, and swelling behaviors of the hydrogels. The findings indicated that raising the hydrazone/imine ratio in the network increased the mechanical properties and stability of the PDH/QCS hybrid hydrogels, owing to the inherent stability of hydrazone bonds. In addition, hydrogels cross-linked with TDA-PEG-FA exhibited superior properties compared to those linked with AA-PEG-FA and SA-PEG-FA due to the presence of hydrophobic domains that functioned as extra physical cross-links in the structure. Besides, P1Q3/TDA-PEG-FA hydrogel with conductive, elastic, and adhesive properties was applied as a wearable device for real-time motion detection. Taken together, this research demonstrated that engineering bonding types and macromeric cross-linkers in the network is a promising approach for developing more efficient and effective hydrogel systems to meet the functional requirements of advanced applications.

## Supplementary Material



## Notes

^1^H NMR and FTIR spectra of synthesized polymers; ninhydrin tests of chitosan and QCS; GPC traces of synthesized polymers; compressive mechanical performance of hydrogels; cyclic compressive stress–strain curves of P1Q3-based hydrogels; photographs of hydrogel before and after tensile loading; tensile mechanical performance of hydrogels; time sweep of hydrogels; images of adhesion test of hydrogels on pig skin; shear strength of P1Q3-based hydrogels; water content of hydrogels; weight remaining of hydrogels; the SEM images and pore sizes of P1Q3-based hydrogels after 7 days of immersion in PBS solution; the images of P1Q3/TDA-PEG-FA hydrogel attached to the skin under various degrees of bending at the finger joint; motion detection of finger joint at 0°, 45°, 90° using P1Q3/TDA-PEG-FA hydrogel; normalized metabolic activities and live/dead staining of MEFs cultured with the hydrogel extract solutions; pore size and porosity of hydrogels analyzed by micro-CT; rheological analyses of hydrogels (PDF)
