# Supplementary material for: Engineering Bonding Types and Macromeric Cross-Linkers in the Network To Modulate the Structures and Properties of Polydextran/Chitosan Hybrid Hydrogels
Source: Biomacromolecules. 2025 Aug 8;26(9):5807–20. doi: 10.1021/acs.biomac.5c00677 (PMC12421511; doi:10.1021/acs.biomac.5c00677)
Supplement: Supplementary file 1 [file bm5c00677_si_001.pdf]

Supporting information

Engineering bonding types and macromeric crosslinkers in the network to modulate the structures and properties of polydextran/chitosan hybrid hydrogels

Pei-Han Lin[a], Tzu-Ying Wang[a], and Yi-Cheun Yeh[a]*

[a] Institute of Polymer Science and Engineering, National Taiwan University, Taipei 10617, Taiwan

*Corresponding authors.

Yi-Cheun Yeh, E-mail: yicheun@ntu.edu.tw

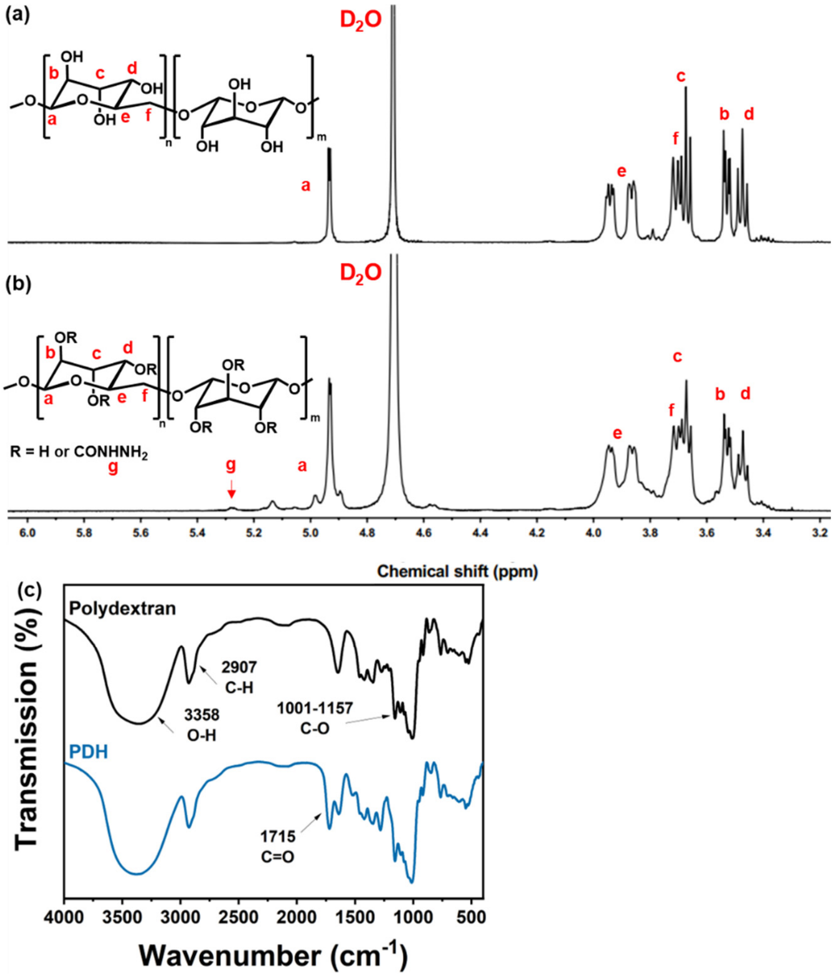

Figure S1. ^{1}H NMR spectra of (a) polydextran and (b) PDH. (c) FTIR spectra of polydextran and PDH.

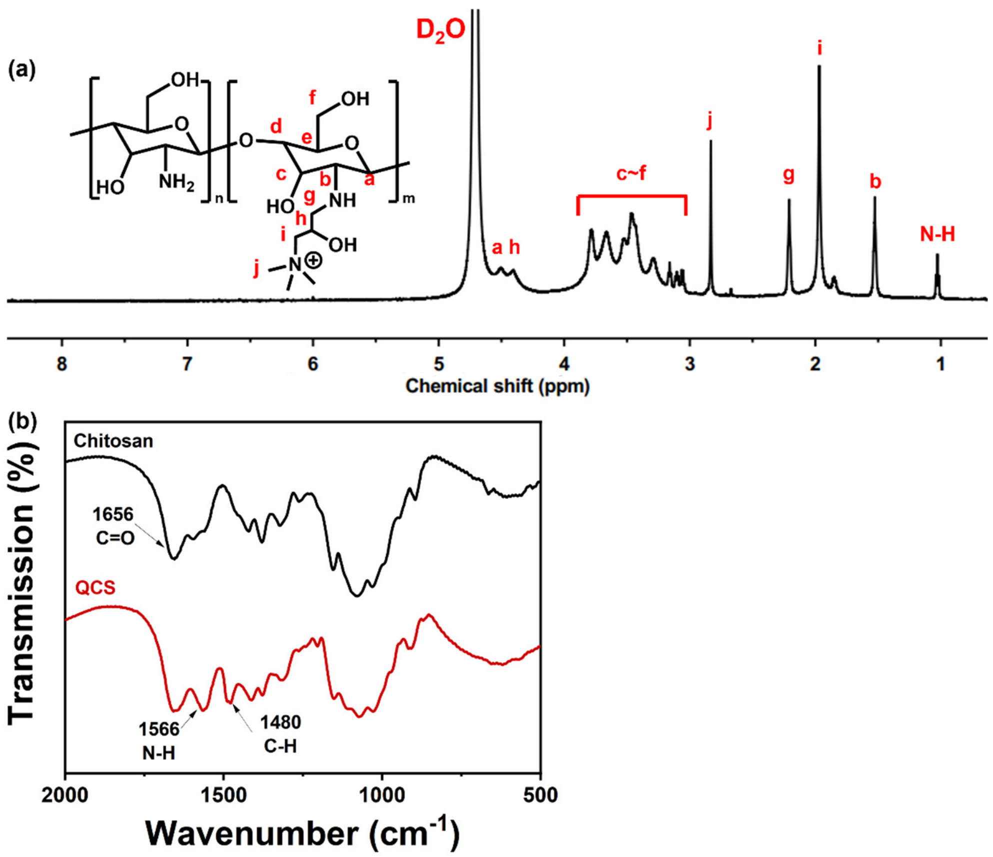

Figure S2. (a) ^{1}H NMR spectrum of QCS. (b) FTIR spectra of chitosan and QCS.

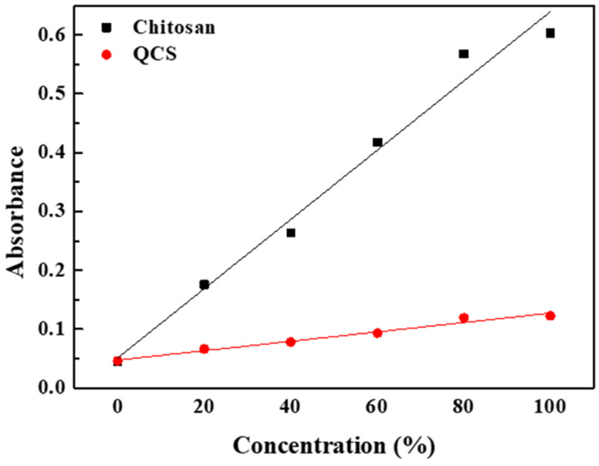

Equation	y = a + b*x		
Weight	No Weighting		
Residual Sum of Squares	0.00425	9.34857E-5	
Adj. R-Square	0.97847	0.97436	
		Value	Standard Error
Chitosan	Intercept	0.05138	0.02359
	Slope	0.00589	3.89592E-4
QCS	Intercept	0.04757	0.0035
	Slope	7.98571E-4	5.77821E-5

Figure S3. Ninhydrin tests of chitosan and QCS.

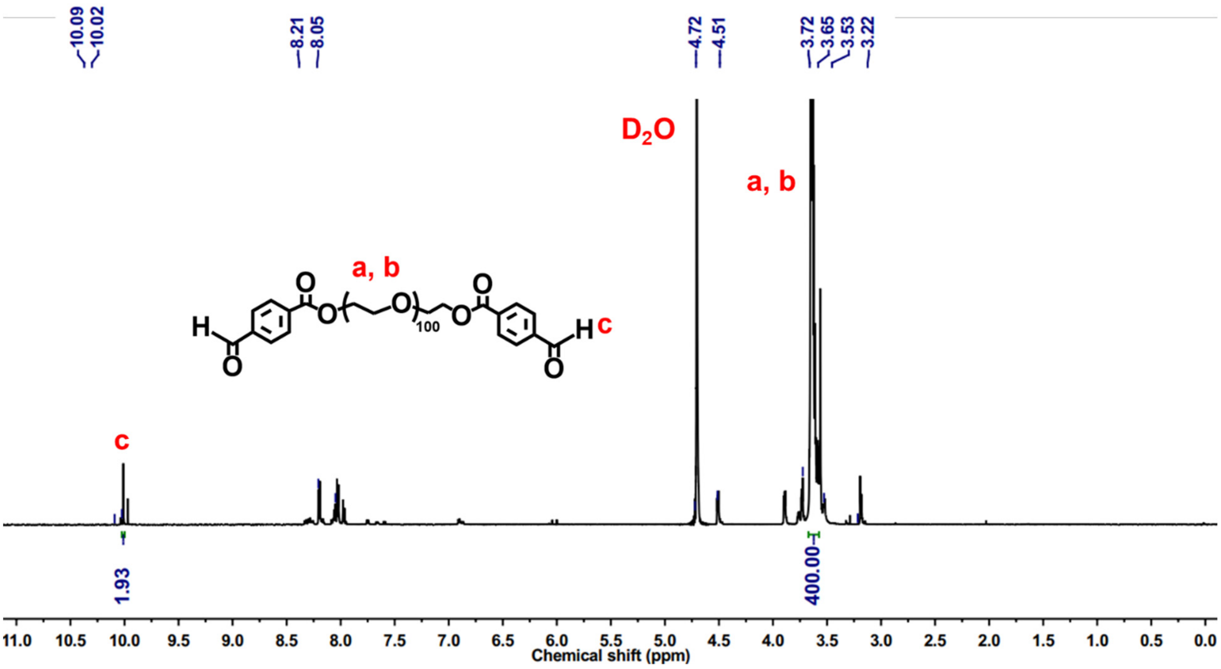

Figure S4. ^{1}H NMR spectrum of PEG-FA.

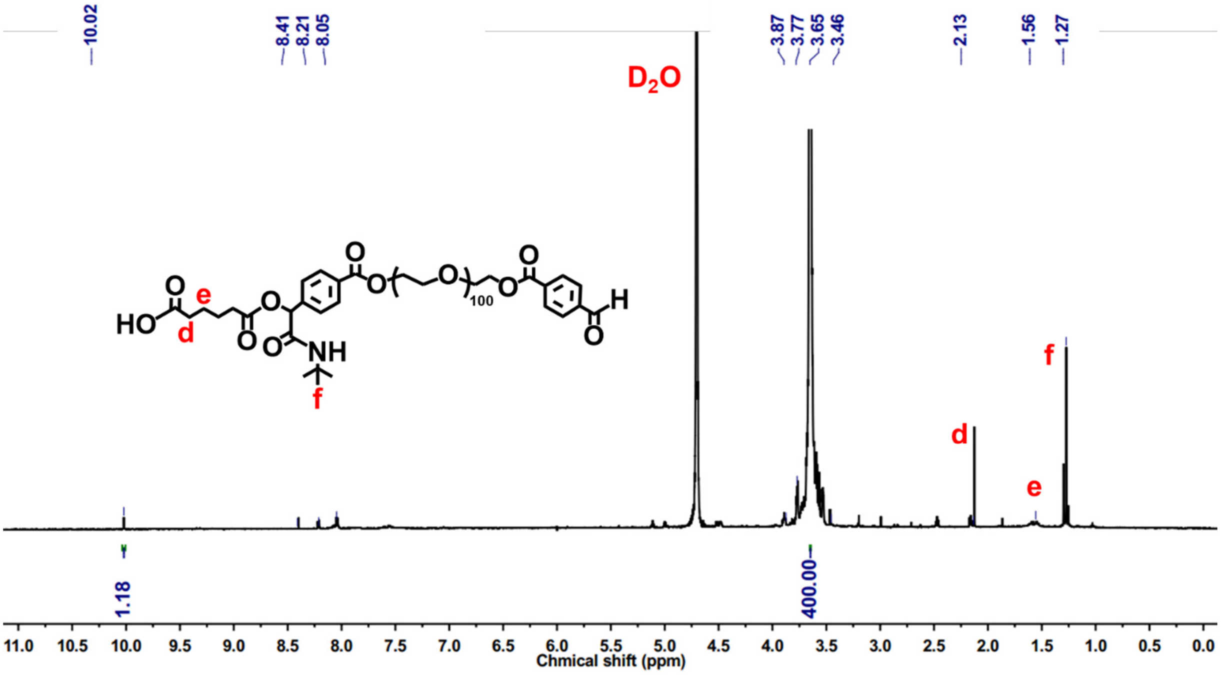

Figure S5. ^{1}H NMR spectrum of AA-PEG-FA.

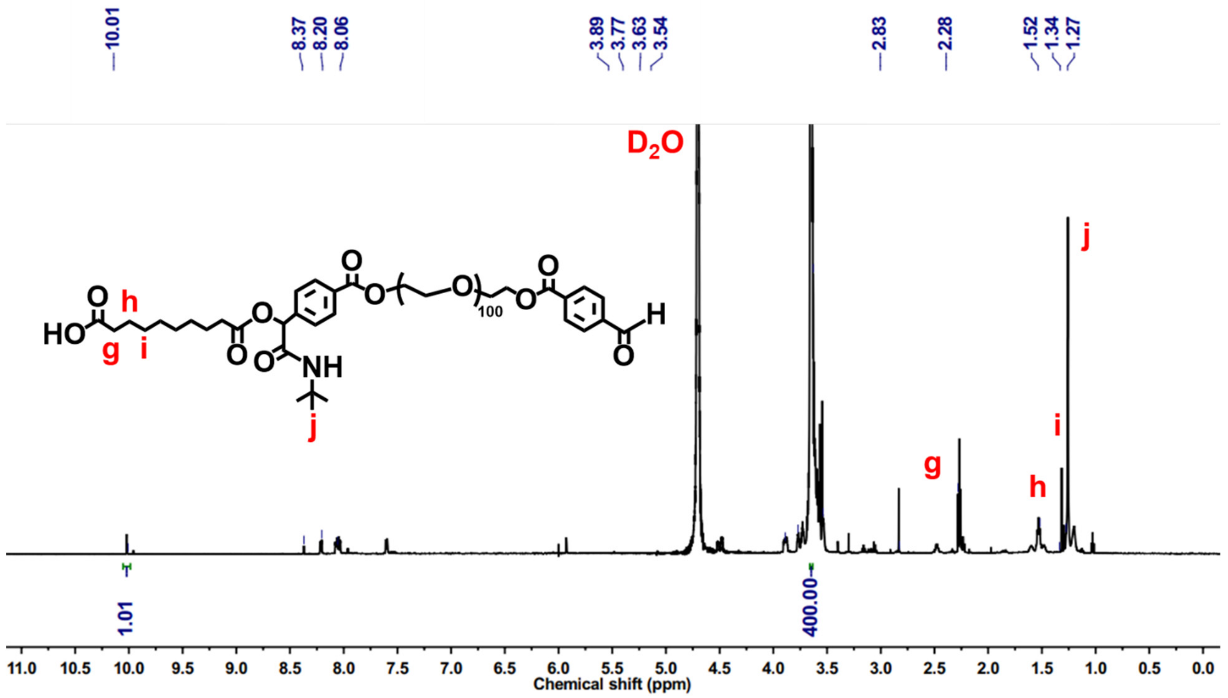

Figure S6. ^{1}H NMR spectrum of SA-PEG-FA.

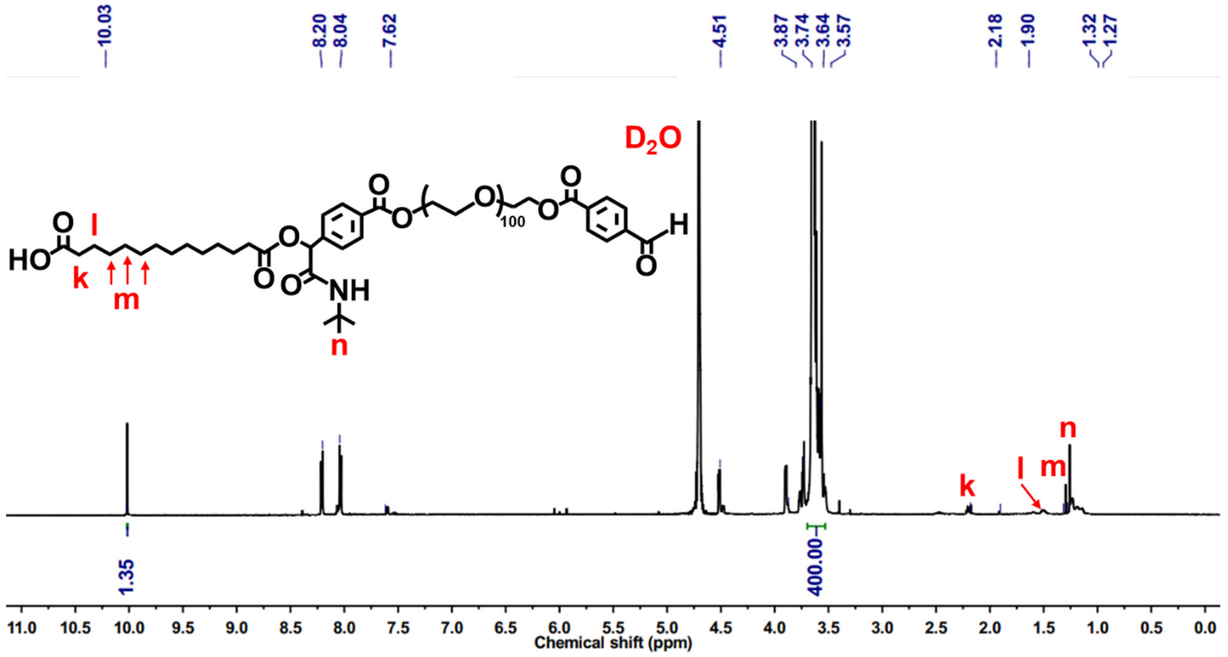

Figure S7. ^{1}H NMR spectrum of TDA-PEG-FA.

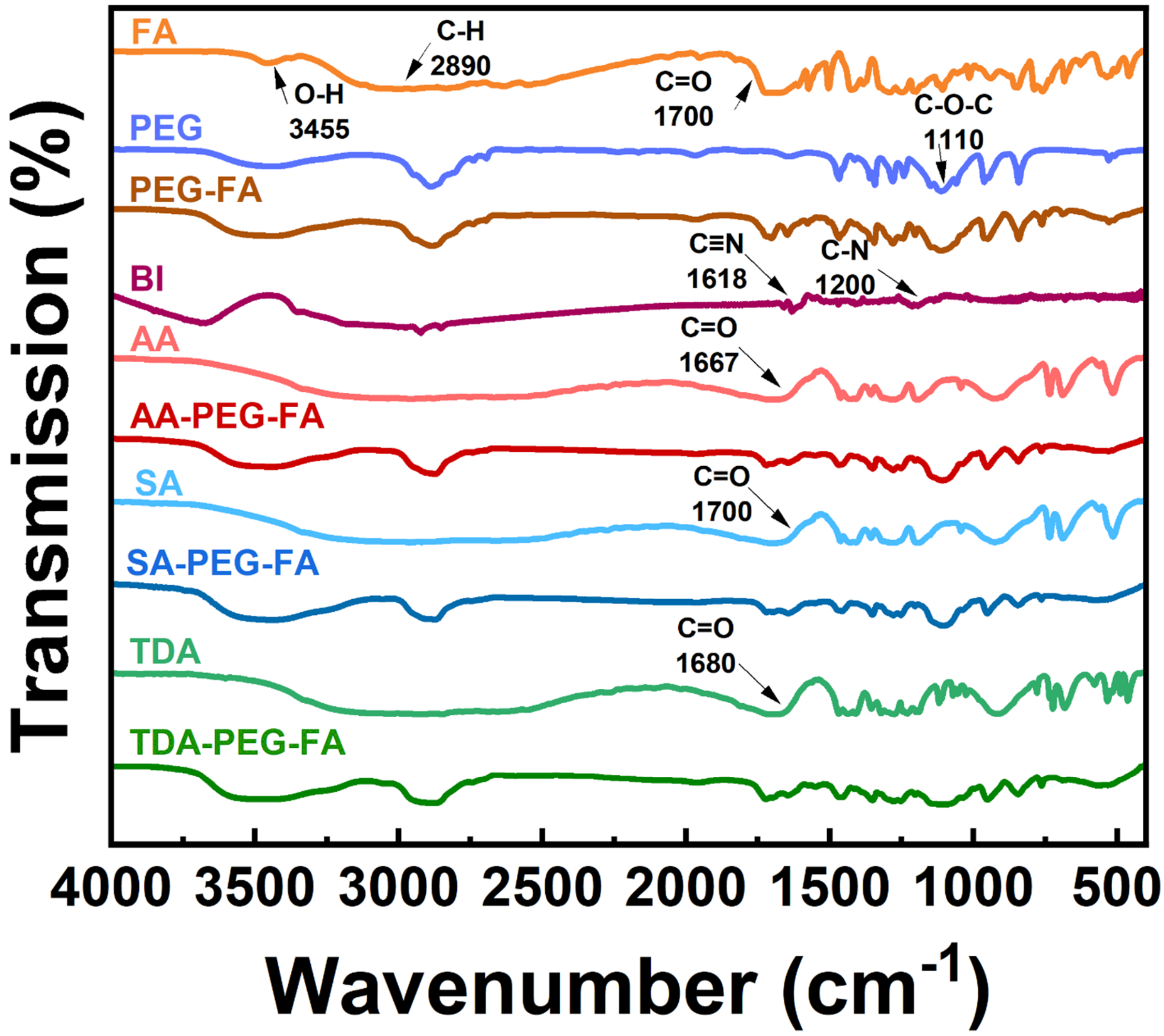

Figure S8. FTIR spectra of molecules and macromers.

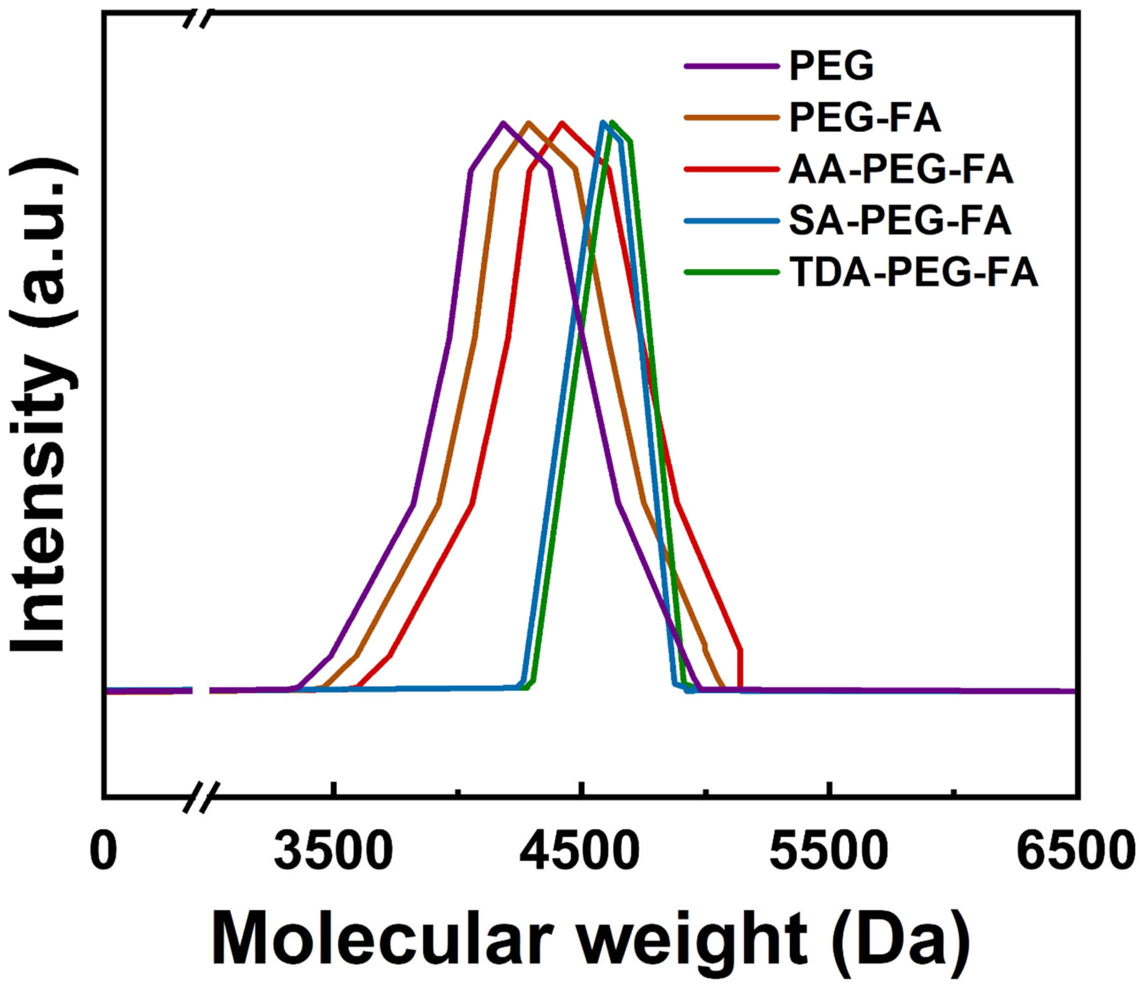

Figure S9. GPC traces of PEG, PEG-FA, AA-PEG-FA, SA-PEG-FA, and TDA-PEG-FA.

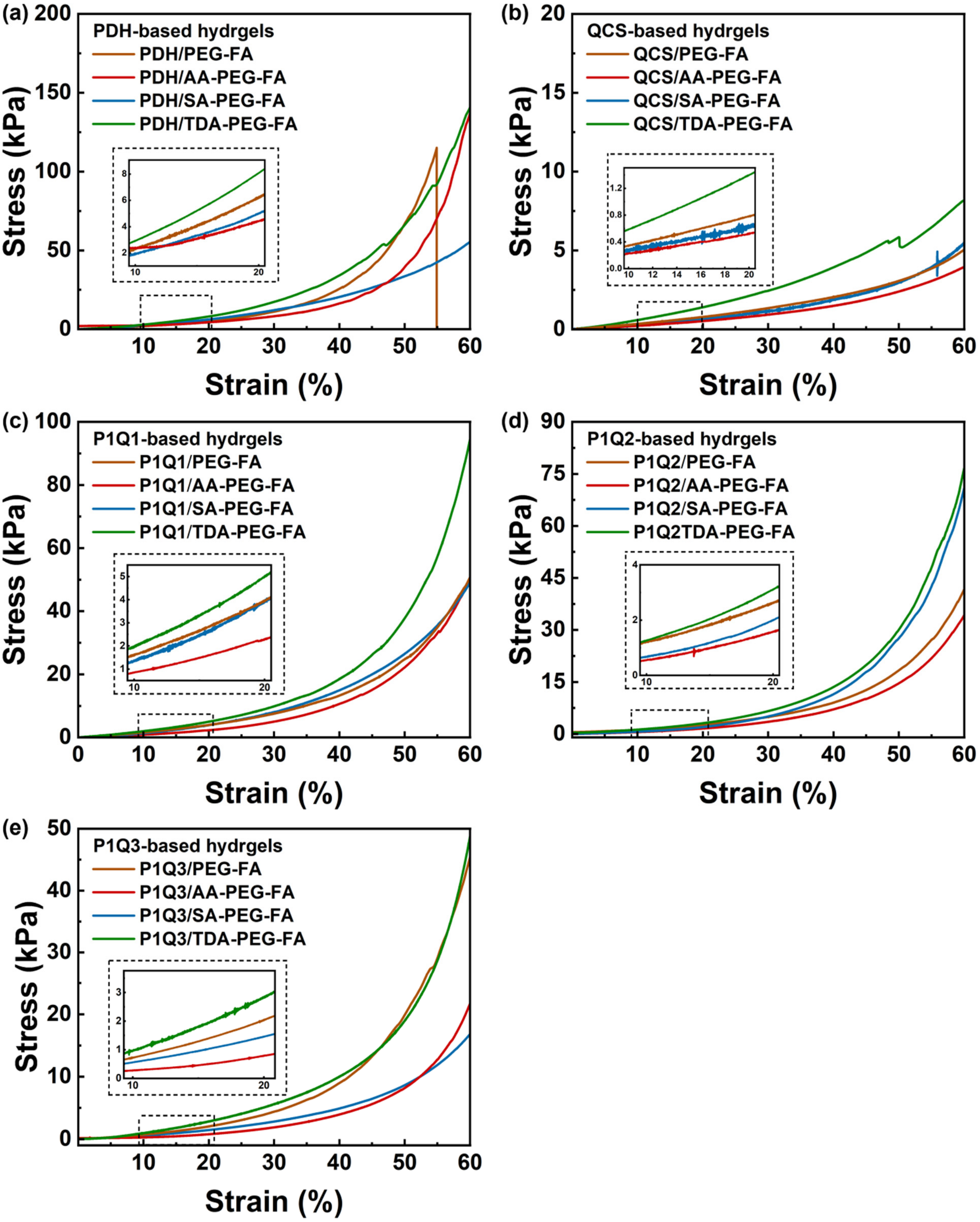

Figure S10. Compressive mechanical performance of (a) PDH-based hydrogels, (b) QCS-based hydrogels, (c) P1Q1-based hydrogels, (d) P1Q2-based hydrogels, (e), and P1Q3-based hydrogels.

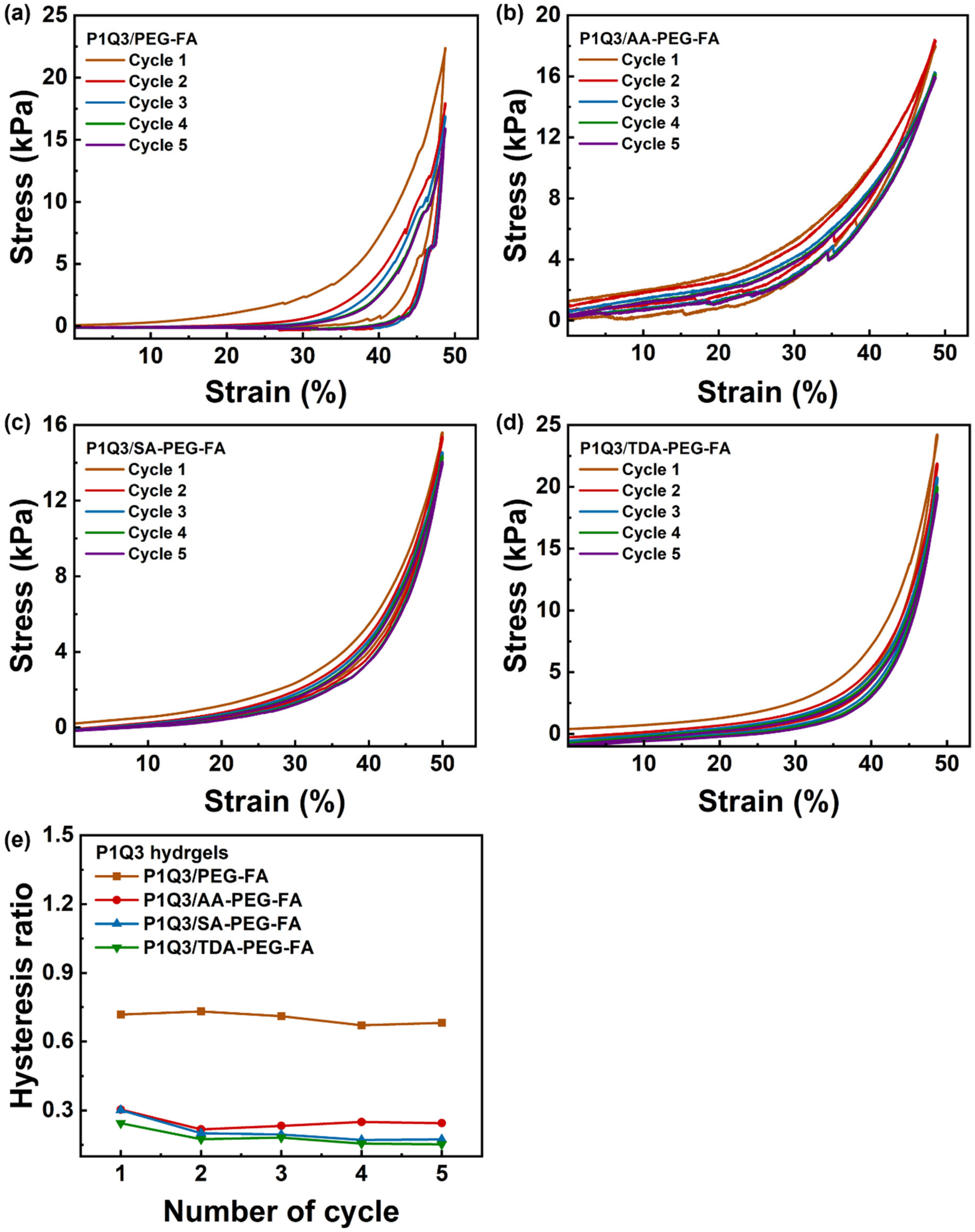

Figure S11. Cyclic compressive stress-strain curves in 5 cycles with a maximum of 50 % strain of (a) P1Q3/PEG-FA, (b) P1Q3/AA-PEG-FA, (c) P1Q3/SA-PEG-FA, and (d) P1Q3/TDA-PEG-FA. (e) Hysteresis ratios of P1Q3-based hydrogels in 5 cycles.

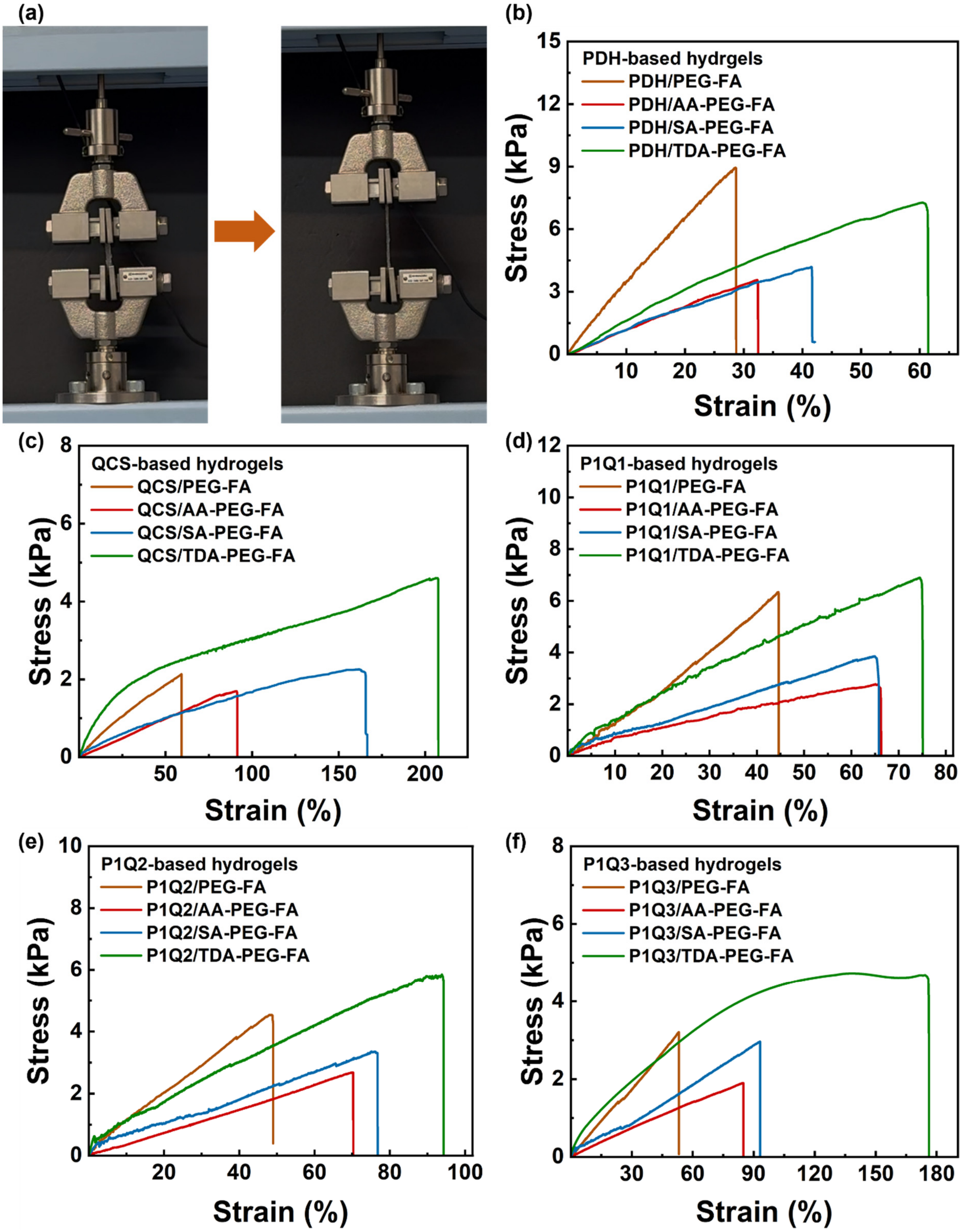

Figure S12. (a) Photographs of hydrogel before and after tensile loading, with images taken immediately before failure. Tensile mechanical performance of (b) PDH-based hydrogels, (c) QCS-based hydrogels, (d) P1Q1-based hydrogels, (e) P1Q2-based hydrogels, and (f) P1Q3-based hydrogels.

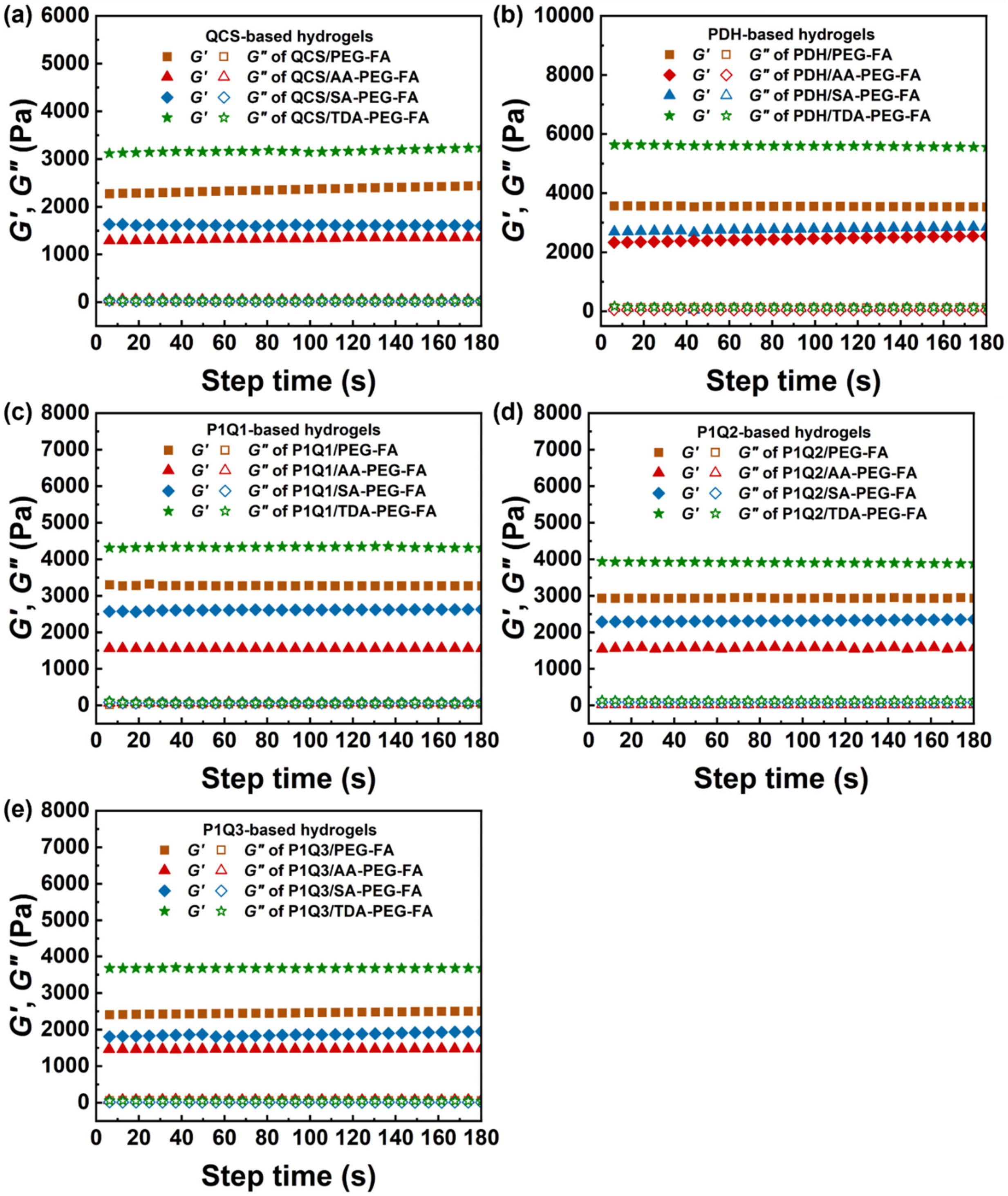

Figure S13. The oscillation time sweep of (a) PDH-based hydrogels, (b) QCS-based hydrogels, (c) P1Q1-based hydrogels, (d) P1Q2-based hydrogels, and (e) P1Q3-based hydrogels.

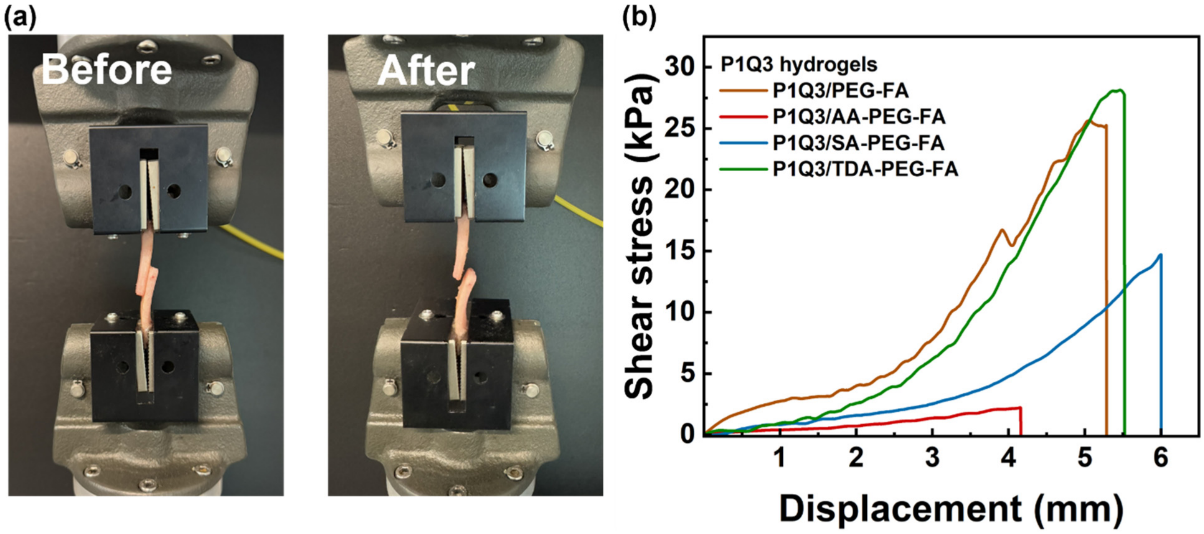

Figure S14. (a) Adhesion test of hydrogels on pig skin. (b) Shear strength of P1Q3-based hydrogels.

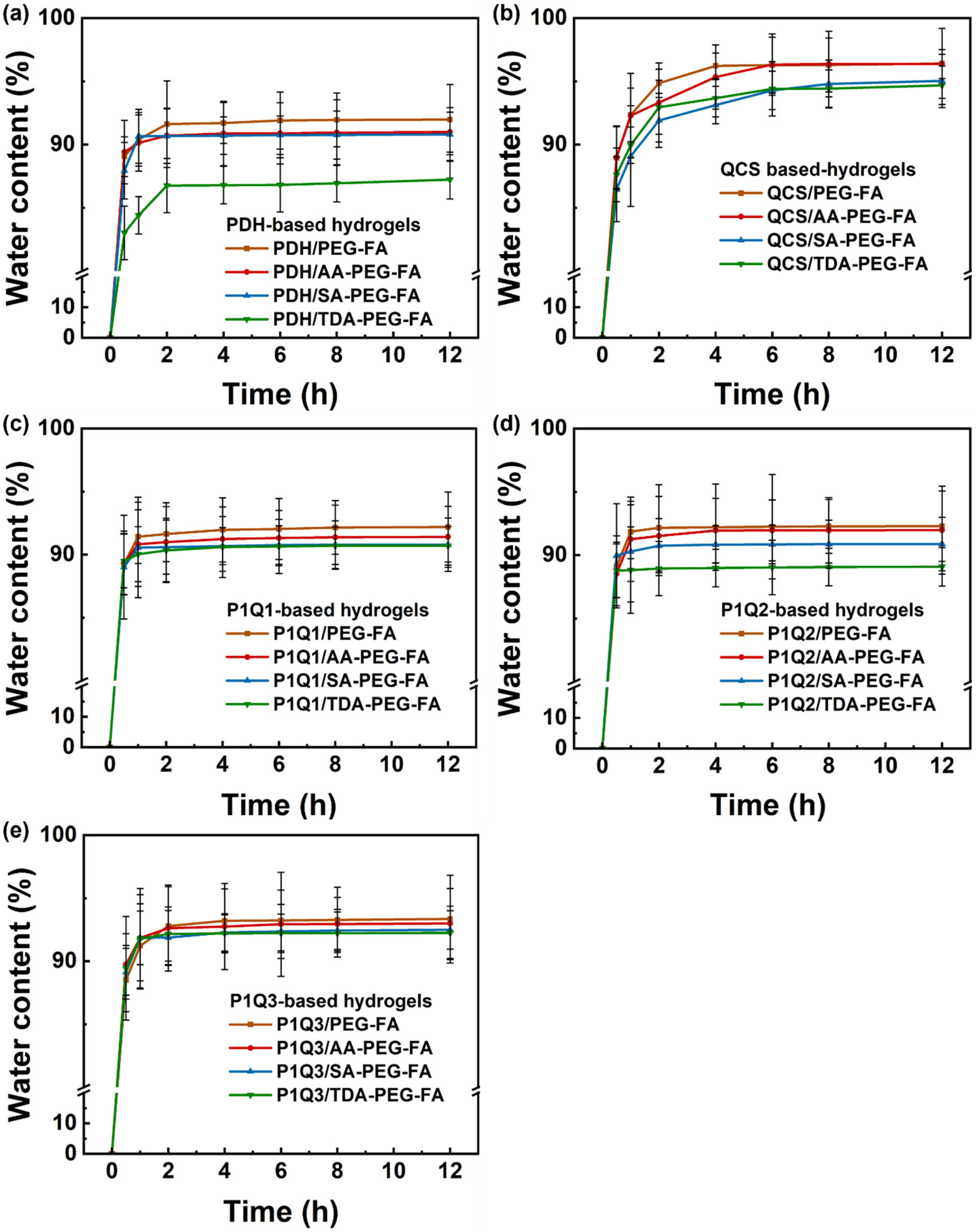

Figure S15. Water content of (a) PDH-based hydrogels, (b) QCS-based hydrogels, (c) P1Q1-based hydrogels, (d) P1Q2-based hydrogels, and (e) P1Q3-based hydrogels.

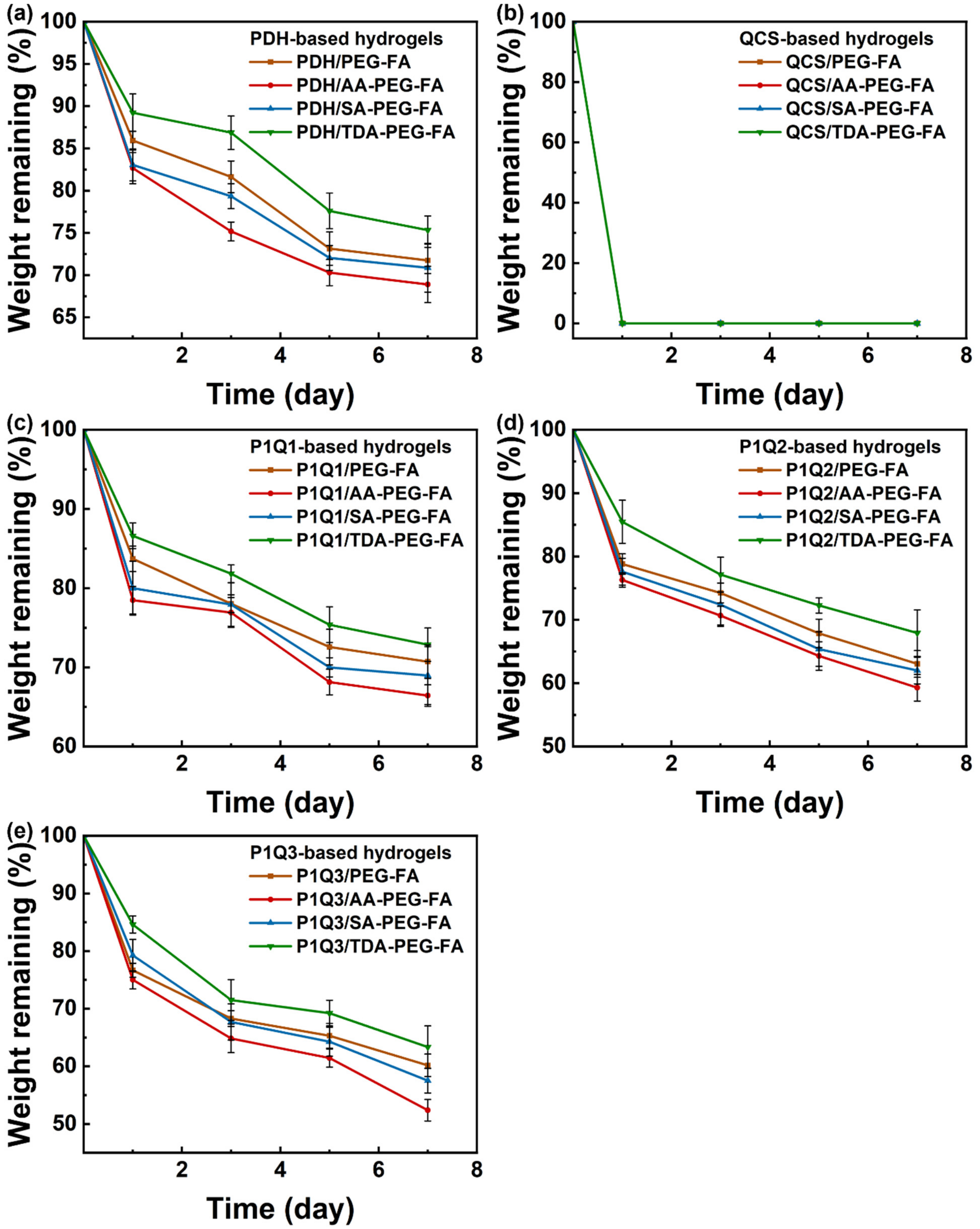

Figure S16. Weight remaining of (a) PDH-based hydrogels, (b) QCS-based hydrogels, (c) P1Q1-based hydrogels, (d) P1Q2-based hydrogels, and (e) P1Q3-based hydrogels.

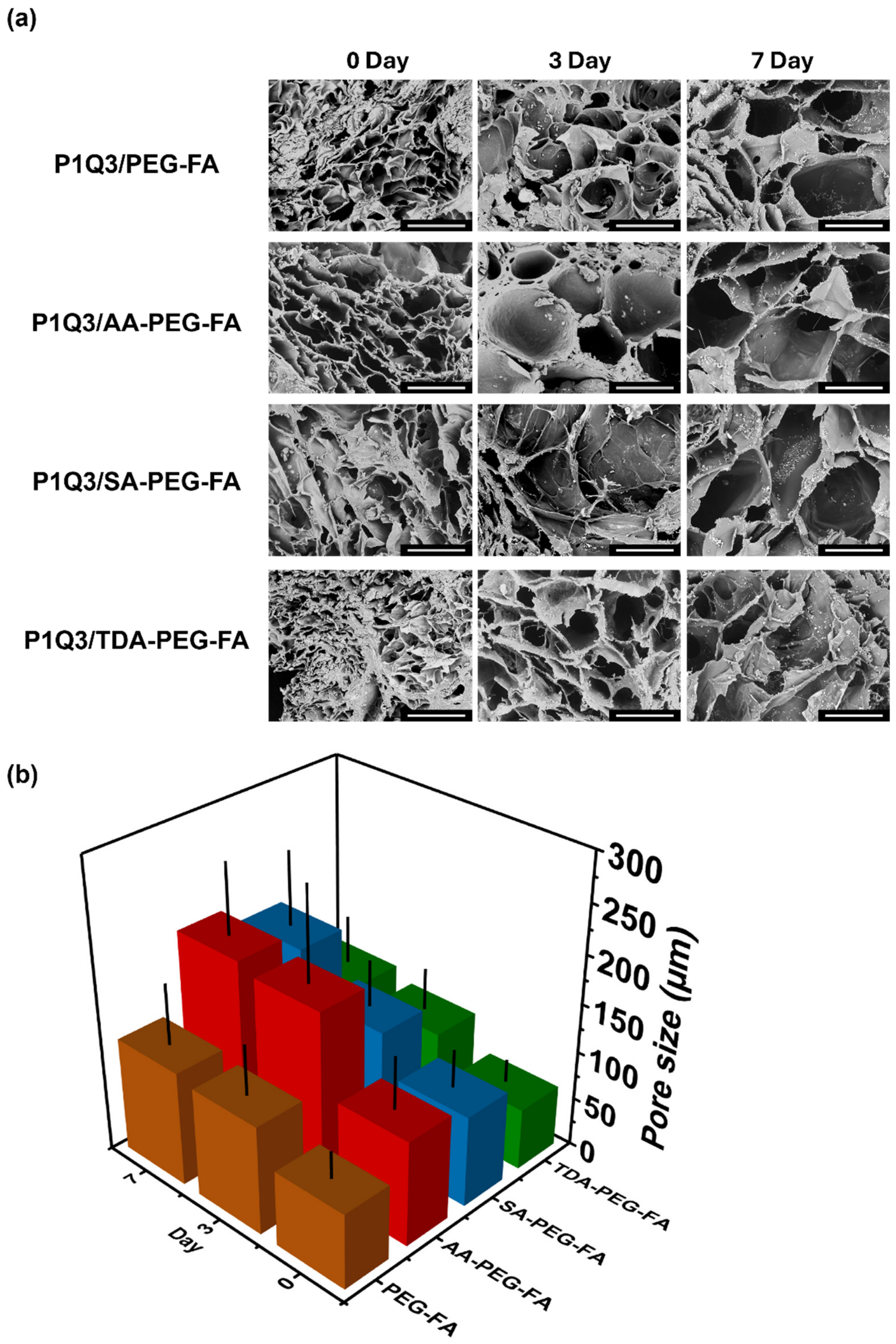

Figure S17 (a) The SEM images. Scale bar: 300 μm, and (b) pore sizes of P1Q3-based hydrogels after 7 days of immersion in PBS solution.

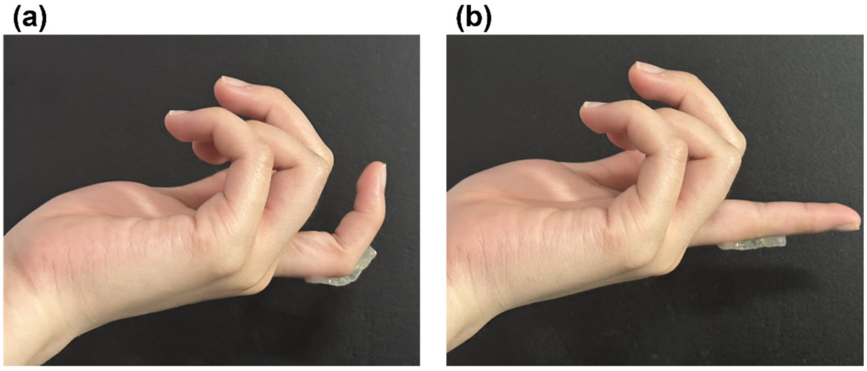

Figure S18. The images of P1Q3/TDA-PEG-FA are attached to the skin under various degrees of bending at the finger joint.

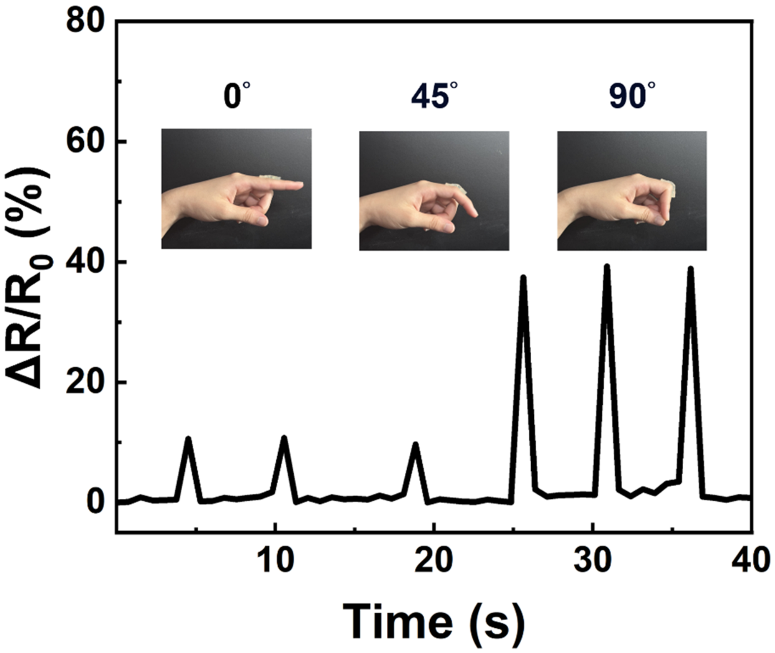

Figure S19. Motion detection of finger joint at 0°, 45°, 90° using P1Q3/TDA-PEG-FA hydrogel.

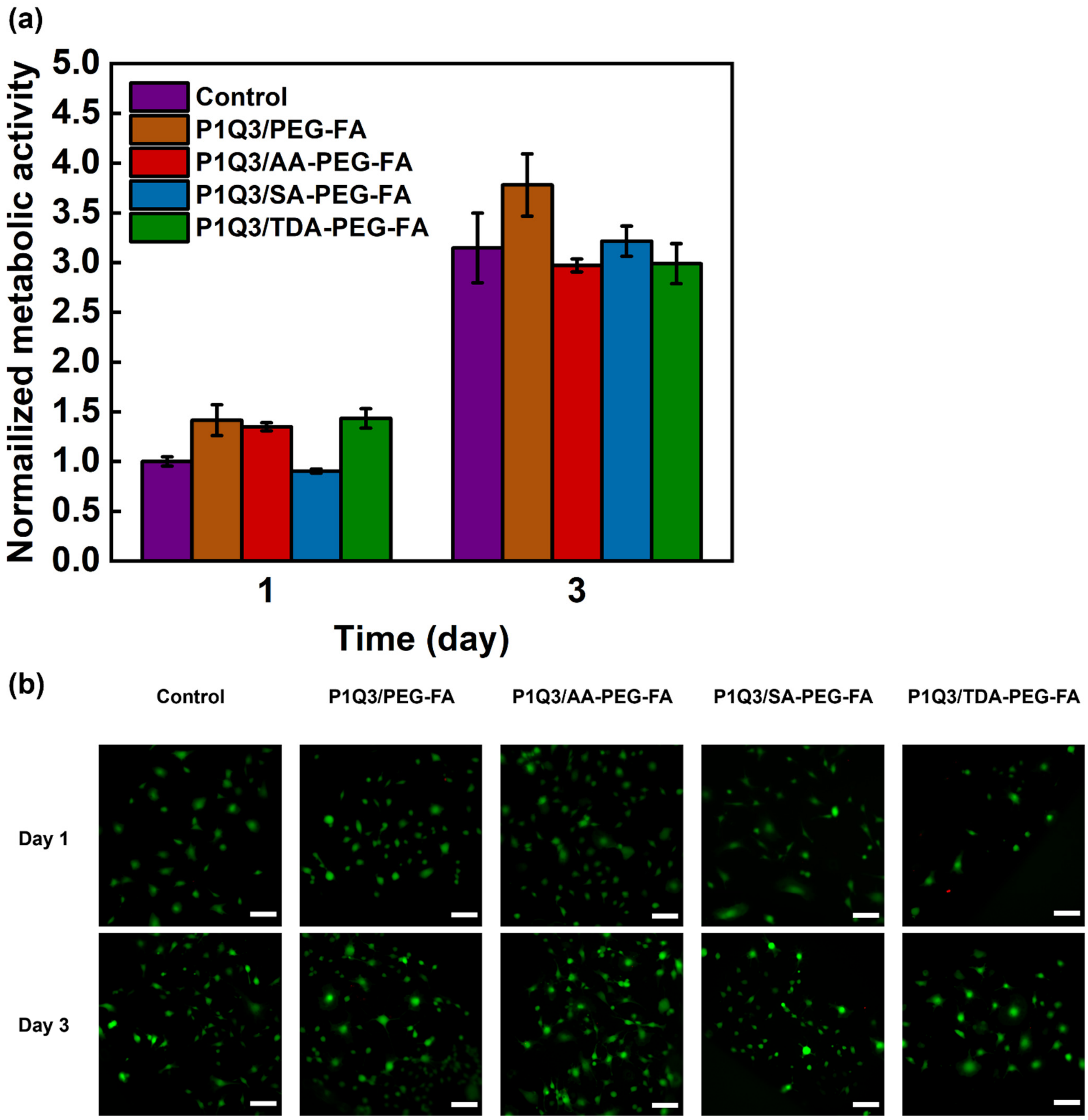

Figure S20. (a) Normalized metabolic activities and (b) live/dead staining of MEFs cultured with P1Q3-based hydrogel extract solutions. The calcein-AM stained live cells green, and the ethidium homodimer-1 selectively stained dead cells red. Scale bar: 100 μm.

Table S1. Pore size and porosity of hydrogels analyzed by micro-CT

	**P1Q3/PEG-FA**	**P1Q3/AA-PEG-FA**	**P1Q3/SA-PEG-FA**	**P1Q3/TDA-PEG-FA**
Average pore size (μm)	90.8	142.3	110.2	90.5
Closed porosity (%)	1.6	0.5	0.1	0.6
Open porosity (%)	40.5	70.7	69.1	64.0
Total porosity (%)	41.4	70.8	69.2	64.3

Table S2. Rheological analyses of hydrogels

	Storage modulus (Pa)	Crosslinking density (mole/m^3)
**PDH/PEG-FA**	3541.33 ± 63.51	1.43 ± 0.03
**PDH/AA-PEG-FA**	2445.67 ± 58.39	0.99 ± 0.02
**PDH/SA-PEG-FA**	2765.45 ± 43.10	1.12 ± 0.02
**PDH/TDA-PEG-FA**	5678.94 ± 80.20	2.29 ± 0.03
**QCS/PEG-FA**	2340.67 ± 14.08	0.94 ± 0.01
**QCS/AA-PEG-FA**	1330.64 ± 25.32	0.53 ± 0.01
**QCS/SA-PEG-FA**	1603.51 ± 21.40	0.65 ± 0.01
**QCS/TDA-PEG-FA**	3133.56 ± 26.17	1.26 ± 0.01
**P1Q1/PEG-FA**	3236.67 ± 68.68	1.32 ± 0.03
**P1Q1/AA-PEG-FA**	1588.39 ± 37.53	0.64 ± 0.02
**P1Q1/SA-PEG-FA**	2602.33 ± 45.54	1.05 ± 0.02
**P1Q1/TDA-PEG-FA**	4323.33 ± 33.08	1.74 ± 0.01
**P1Q2/PEG-FA**	2984.67 ± 46.31	1.20 ±0.02
**P1Q2/AA-PEG-FA**	1547.33 ± 33.19	0.62 ± 0.01
**P1Q2/SA-PEG-FA**	2311.64 ± 26.13	0.93 ± 0.01
**P1Q2/TDA-PEG-FA**	3952.65 ± 37.66	1.59 ± 0.01
**P1Q3/PEG-FA**	2516.37 ± 54.78	1.01 ± 0.02
**P1Q3/AA-PEG-FA**	1424.35 ± 30.47	0.57 ± 0.01
**P1Q3/SA-PEG-FA**	1868.67 ± 30.73	0.75 ± 0.01
**P1Q3/TDA-PEG-FA**	3613.56 ± 50.46	1.46 ± 0.02
